# Phase variation of a signal transduction system controls *Clostridioides difficile* colony morphology, motility, and virulence

**DOI:** 10.1371/journal.pbio.3000379

**Published:** 2019-10-28

**Authors:** Elizabeth M. Garrett, Ognjen Sekulovic, Daniela Wetzel, Joshua B. Jones, Adrianne N. Edwards, Germán Vargas-Cuebas, Shonna M. McBride, Rita Tamayo

**Affiliations:** 1 Department of Microbiology and Immunology, University of North Carolina Chapel Hill, Chapel Hill, North Carolina, United States of America; 2 Department of Molecular Biology and Microbiology, Tufts University School of Medicine, Boston, Massachusetts, United States of America; 3 Department of Microbiology and Immunology, Emory University, Rollins Research Center, Atlanta, Georgia, United States of America; Swiss Federal Institute of Technology Lausanne (EPFL), SWITZERLAND

## Abstract

Recent work has revealed that *Clostridioides difficile*, a major cause of nosocomial diarrheal disease, exhibits phenotypic heterogeneity within a clonal population as a result of phase variation. Many *C*. *difficile* strains representing multiple ribotypes develop two colony morphotypes, termed rough and smooth, but the biological implications of this phenomenon have not been explored. Here, we examine the molecular basis and physiological relevance of the distinct colony morphotypes produced by this bacterium. We show that *C*. *difficile* reversibly differentiates into rough and smooth colony morphologies and that bacteria derived from the isolates display discrete motility behaviors. We identified an atypical phase-variable signal transduction system consisting of a histidine kinase and two response regulators, named herein colony morphology regulators RST (CmrRST), which mediates the switch in colony morphology and motility behaviors. The CmrRST-regulated surface motility is independent of flagella and type IV pili, suggesting a novel mechanism of cell migration in *C*. *difficile*. Microscopic analysis of cell and colony structure indicates that CmrRST promotes the formation of elongated bacteria arranged in bundled chains, which may contribute to bacterial migration on surfaces. In a hamster model of acute *C*. *difficile* disease, the CmrRST system is required for disease development. Furthermore, we provide evidence that CmrRST phase varies during infection, suggesting that the intestinal environment impacts the proportion of CmrRST-expressing *C*. *difficile*. Our findings indicate that *C*. *difficile* employs phase variation of the CmrRST signal transduction system to generate phenotypic heterogeneity during infection, with concomitant effects on bacterial physiology and pathogenesis.

## Abstract 2

In many bacterial species, a genetically clonal population can generate phenotypic heterogeneity to ensure survival of a subpopulation in the face of sudden environmental stressors. This work shows that the intestinal pathogen Clostridioides difficile introduces heterogeneity into the population through the phase-variable expression of a signal transduction system, resulting in broad changes in physiology, motility, and virulence.

## Introduction

Phenotypic heterogeneity within bacterial populations is a widely established phenomenon that allows the population to survive sudden environmental changes [1–3]. Heterogeneity serves as a “bet-hedging” strategy such that a subpopulation can persist and propagate an advantageous phenotype. Phase variation is one mechanism that imparts phenotypic heterogeneity, typically by controlling the ON/OFF production of a surface-exposed factor that directly interfaces with the environment [4,5]. Many human pathogens, including pathogenic *Escherichia coli*, *Neisseria meningitidis*, and *Streptococcus pneumoniae*, employ phase variation to evade the host immune system and increase host colonization, persistence, and virulence [4,6–13]. Phase-variable phenotypes are heritable yet reversible, allowing the surviving subpopulation to regenerate heterogeneity. In phase variation by conservative site-specific recombination, a serine or tyrosine recombinase mediates the inversion of a DNA sequence adjacent to the regulated gene(s) [5]. The invertible DNA sequences are flanked by inverted repeats and contain the regulatory information, such as a promoter, that when properly oriented controls gene expression in cis.

*Clostridioides difficile* (formerly *Clostridium difficile*) is a gram-positive, spore-forming, obligate anaerobe and a significant public health burden globally, causing gastrointestinal disease ranging from diarrhea to potentially fatal complications such as pseudomembranous colitis, toxic megacolon, bowel perforation, and sepsis. *C*. *difficile* pathogenesis is primarily driven by the toxins TcdA and TcdB, which inactivate host Ras homologous (Rho)-family guanosine triphosphatases (GTPases), resulting in actin cytoskeleton dysregulation, tight junction disruption, and host cell death; consequently, TcdA and TcdB compromise the epithelial barrier and elicit diarrheal symptoms and inflammation [14–16]. Many aspects of *C*. *difficile* physiology and pathogenesis remain poorly understood. For example, some *Clostridioides* species, including *C*. *difficile*, are capable of forming two distinct colony morphologies; one is smooth and circular, and the other is rough and filamentous [17–20]. However, the underlying mechanisms and physiological relevance of this dimorphism are unknown. Many bacterial species develop multiple colony morphologies as a result of the regulated production of surface factors, which can impact diverse physiological traits and behaviors [21–26]. In multiple species, the production of surface factors is subject to phase variation, leading to changes in gross colony morphology. In *S*. *pneumoniae* and *Acinetobacter baumannii*, phase variation of capsule polysaccharides leads to the formation of either opaque or translucent colonies that differ in a multitude of phenotypes, including cell morphology, biofilm formation, antibiotic sensitivity, host colonization, and virulence [6,9,12,24,26,27]. Phase variation of colony morphology is therefore an important adaptive strategy during infection for multiple pathogens.

The biological significance and mechanisms underlying colony morphology development of *C*. *difficile* have not been reported. *C*. *difficile* encodes multiple factors that are regulated through phase variation. In *C*. *difficile*, eight invertible DNA sequences, or “switches,” have been identified, seven of which are present in the epidemic strain R20291 [28,29]. Two have been characterized: one controlling phase variation of cell wall protein V (CwpV) and the other controlling flagellar phase variation [19,30–32]. The phase-variable *flgB* flagellar operon encodes sigma factor D (SigD), which coordinates flagellar gene expression and positively regulates the *tcdA* and *tcdB* toxin genes [33–35]. Consequently, the flagellar switch mediates phase variation of flagella and toxin production, highlighting the potential impact of phase variation on *C*. *difficile* physiology and virulence.

As in many bacteria, the intracellular signaling molecule cyclic diguanosine monophosphate (c-di-GMP) regulates the transition between community-associated and planktonic, motile lifestyles of *C*. *difficile* [34,36–39]. This regulation occurs in part through direct control of gene expression by c-di-GMP via riboswitches [40–43]. For example, a c-di-GMP riboswitch lies in the 5′ leader sequence of the *flgB* operon and causes transcription termination when c-di-GMP is bound, thus inhibiting swimming motility [40,41,44]. Because flagellum and toxin gene expression is linked through SigD, c-di-GMP also inhibits toxin production [35,44]. Conversely, a c-di-GMP riboswitch upstream of the type IV pilus (TFP) locus allows c-di-GMP to positively regulate gene expression and promote TFP-dependent behaviors such as autoaggregation, surface motility, biofilm formation, and colonization of host tissues [34,36–38,44,45]. Additionally, c-di-GMP regulates the expression and cell wall anchoring of surface proteins and adhesins [42,46,47]. Therefore, c-di-GMP coordinates the expression of multiple surface factors with implications for pathogenesis.

In this study, we characterized rough and smooth colony isolates of *C*. *difficile* R20291 and determined that they exhibit distinct motility behaviors in vitro. Colony morphology and associated motility phenotypes are controlled by both phase variation and c-di-GMP, and colony morphology is independent of TFP and flagella. We identified the c-di-GMP-regulated, phase-variable signal transduction system, consisting of a putative histidine kinase and two DNA-binding response regulators, that modulates colony morphology, surface migration, and swimming motility. Finally, we provide evidence using a hamster model of infection for phase variation and a role for the colony morphology regulators RST (CmrRST) system in virulence. The link between phase variation and c-di-GMP signaling to control CmrRST production suggests a mechanism for switching a global expression program during infection, which appears to have critical implications for disease development.

## Results

### *C*. *difficile* strains of diverse ribotypes develop two distinct, phase-variable colony morphotypes

**Fig 1:**
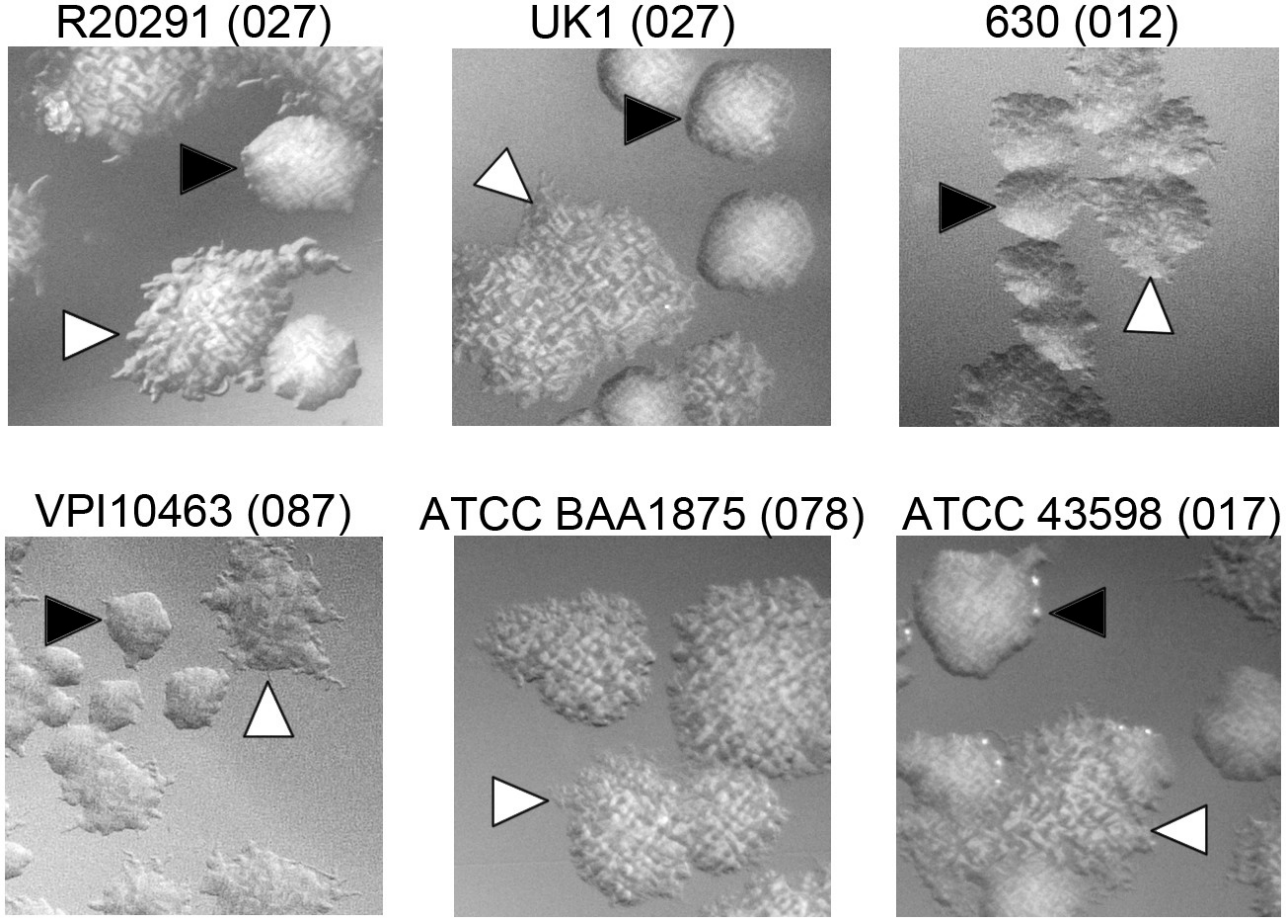
Formation of rough and smooth colonies by multiple *C*. *difficile* strains. *C*. *difficile* strains representing five different ribotypes (indicated in parentheses) were grown on BHIS agar medium for 72 hours to allow differentiation of colony morphology. Growth was recovered and plated on BHIS agar for visualization of individual colonies. Shown are representative images from two independent experiments. Black triangles indicate smooth colonies, and white triangles indicate rough colonies. All images were taken at ×2 magnification. ATCC, American Type Culture Collection; BHIS, brain heart infusion plus yeast.

The *C*. *difficile* strain R20291, a ribotype 027 strain associated with epidemic infections, exhibits two distinct colony morphologies: a smooth colony that is round and circular and a rough colony that is flatter and has filamentous edges [18–20]. In addition to R20291, strains UK1 (ribotype 027), VPI10463 (ribotype 087), 630 (ribotype 012), and American Type Culture Collection (ATCC) 43598 (ribotype 017) yielded both rough and smooth colonies (Fig 1). Some of these strains (R20291 and UK1) showed both colony morphotypes through routine plating, whereas others (630) required extended incubation to allow the appearance of bacteria that develop rough colonies. ATCC BAA 1875 (ribotype 078) did not develop smooth colonies under any conditions tested. Therefore, many strains are capable of generating two distinct colony morphologies in vitro.

**Fig 2:**
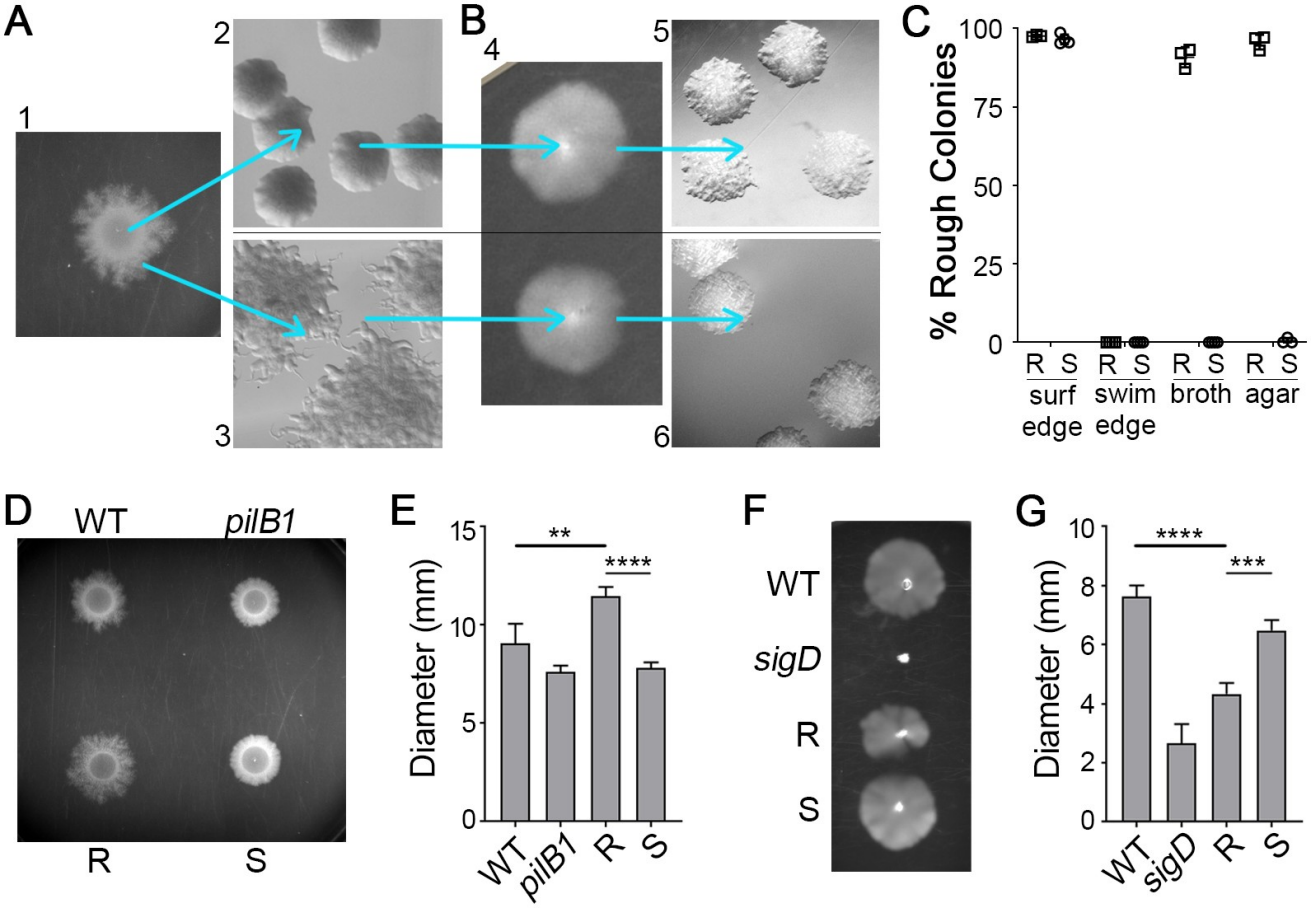
Reversible selection of distinct colony morphotypes with opposing motility phenotypes. (A) *C*. *difficile* R20291 liquid culture was spotted on BHIS-1.8% agar and grown for 48 hours to allow spreading growth (panel 1). Bacteria collected from the center of a spot yield mostly smooth colonies (panel 2), whereas bacteria from the edge yield almost exclusively rough colonies (panel 3). (B) Rough and smooth colony isolates obtained as in (A) were passaged in 0.5× BHIS-0.3% agar (panel 4), and samples were collected from the edge of motile growth. Both rough and smooth colony isolates only gave rise to smooth colonies (panels 5 and 6). Shown are representative images of colonies from four independent experiments. All images were taken at ×2 magnification. (C) Quantification of colony morphology for rough and smooth colony isolates after passage on a BHIS agar surface (surf) as in (A) or in motility medium (swim) as in (B), with samples collected from the edges of growth. R, S indicate rough and smooth starting inocula, respectively. Rough and smooth isolates were also directly passaged through BHIS broth medium or struck on a BHIS agar plate and then enumerated. Symbols indicate individual values from three to four independent experiments, and bars indicate means and standard deviations. (D, E) *C*. *difficile* R20291, a TFP-null control (*pilB1*), and rough and smooth colony isolates were assayed for surface motility on BHIS-1.8% agar. (D) A representative of four experiments is shown. (E) Surface motility was quantified by measuring the diameter of growth after 48 hours. (F, G) *C*. *difficile* R20291, a nonmotile control (*sigD*), and rough and smooth colony isolates were assayed for swimming motility in 0.5× BHIS-0.3% agar. (F) A representative of four experiments is shown. (G) Swimming motility was quantified by measuring the diameter of growth after 48 hours. (E,G) **p < 0.01, ***p < 0.001, ****p < 0.0001, one-way ANOVA and Tukey’s posttest. Data can be found in supplemental file S1 Data. BHIS, brain heart infusion plus yeast; R, rough; S, smooth; *sigD*, sigma factor D gene; surf, surface; TFP, type IV pilus; WT, wild-type.

We and others previously observed that spotting a culture of *C*. *difficile* R20291 on an agar surface results in expansion of the colony as dendritic, filamentous growth [18,34]. We discovered that this culturing method allows the segregation of the two colony morphologies. Streaking from the center of the colony yielded a population of predominantly smooth colonies, whereas streaking from the edges yielded a population of predominantly rough colonies (Fig 2A). To evaluate the stability of the colony phenotypes, we passaged rough and smooth colonies through multiple growth conditions. Streaking a rough or smooth colony again onto agar medium resulted in overall maintenance of the original morphology (Fig 2A and 2C), indicating that colony morphology is heritable and generally stable under specific growth conditions. Similarly, the starting morphology was maintained when the isolates were passaged in broth (Fig 2C). Because surface growth promoted the development of the rough morphotype, we speculated that conditions favoring swimming motility might yield smooth colonies. Rough and smooth colonies were inoculated into soft (0.3%) agar to allow for swimming motility over 48 hours (Fig 2B, panel 4). Growth recovered from the edges of the motile growth solely yielded smooth colonies, regardless of the starting morphology, indicating that colony morphology is reversible (Fig 2B and 2C). These data support that *C*. *difficile* colony morphology phase varies and reveal in vitro growth conditions that select for a specific phase variant: expansion of a colony on an agar surface favors bacteria that yield rough colonies, whereas swimming conditions select for bacteria that yield exclusively smooth colonies.

### Rough and smooth colony isolates exhibit distinct motility behaviors

The observation that surface growth favors bacteria that develop rough colonies suggests that the rough morphotype has an advantage over smooth in this growth condition. Conversely, smooth morphotype bacteria may have an advantage over the rough in swimming motility. To test this, we assessed the motility behaviors of rough and smooth colony isolates. Expansion of colonies of “wild-type” (WT) (i.e., undifferentiated by colony morphology) R20291 on an agar surface is characterized by initial (approximately 24 hours) growth restricted to the site of inoculation and then spreading tendrils of growth between 24 and 96 hours [37]. Type IV pili were previously shown to contribute to motility on an agar surface, so the nonpiliated *pilB1* mutant lacking the PilB ATPase required for TFP assembly was included as a control [36,37]. In this assay, bacteria isolated from rough colonies exhibited greater colony expansion after 48 hours compared with undifferentiated (WT) R20291 (Fig 2D and 2E). Conversely, bacteria derived from smooth colonies were deficient in colony expansion compared with the rough isolates and more similar to the *pilB1* control after 48 hours (Fig 2D and 2E), though the smooth isolates remained capable of colony expansion.

Flagellum-dependent swimming motility was assayed by inoculating bacteria into 0.3% agar and measuring migration through the medium. Undifferentiated R20291 and a nonmotile *sigD* mutant served as controls. Rough colony isolates showed significantly decreased swimming motility compared with smooth and undifferentiated populations (Fig 2F and 2G). Bacteria from smooth colonies showed swimming motility comparable to the undifferentiated parent (Fig 2F and 2G), likely because the parental isolate consisted primarily of bacteria that yield smooth colonies. These results indicate that rough and smooth colony morphotypes have distinct motility behaviors.

### Colony morphology is regulated by a phase-variable signal transduction system and c-di-GMP

**Fig 3:**
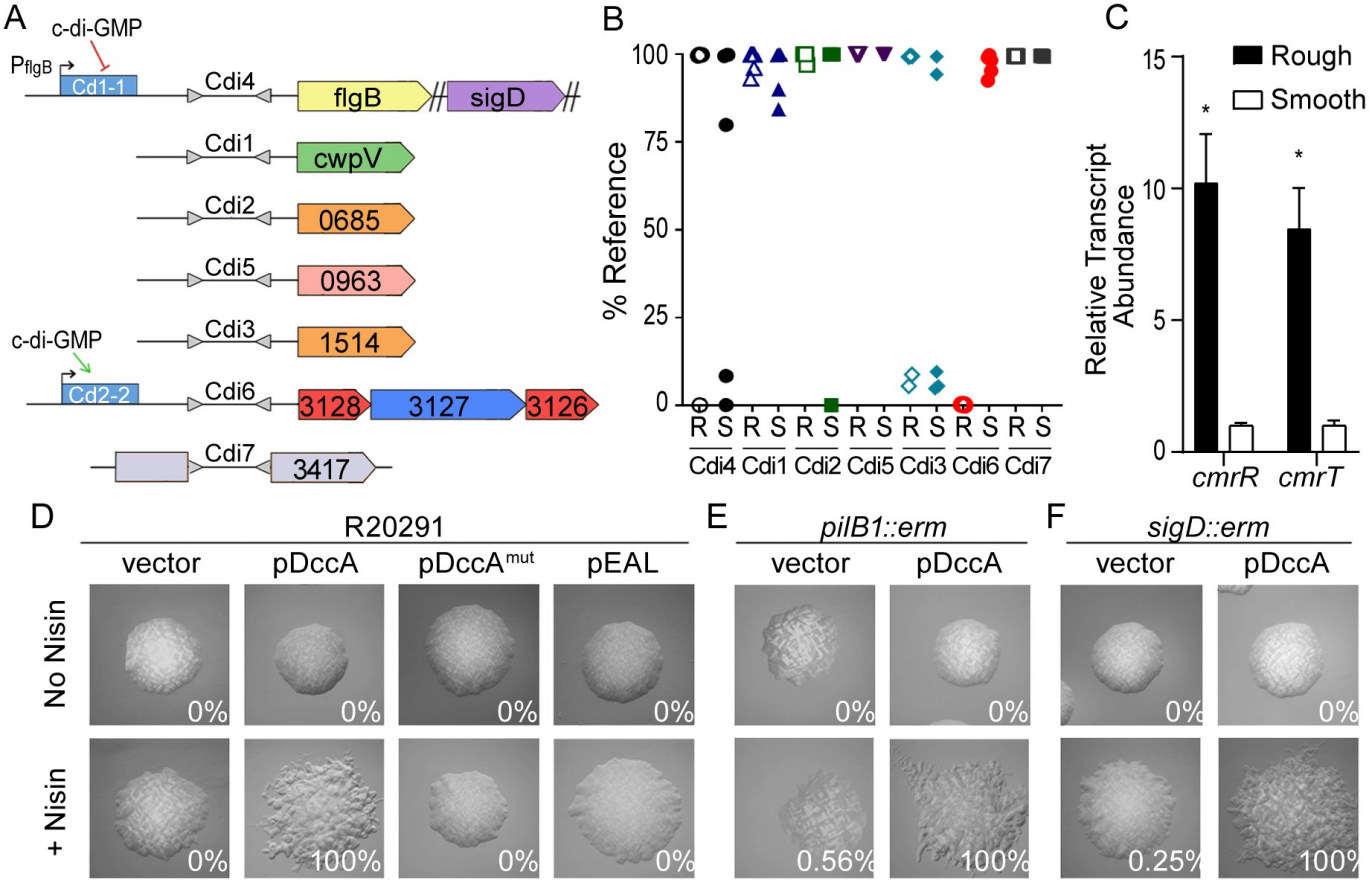
Expression of a c-di-GMP regulated phosphorelay system promotes rough colony formation in a TFP- and flagellum-independent manner. (A) Diagram of seven invertible DNA elements previously identified in *C*. *difficile* R20291. Gray triangles represent inverted repeats flanking invertible elements, which are named according to previously defined nomenclature [29]. Downstream regulated genes are shown. Blue rectangles denote c-di-GMP riboswitches, and direction of regulation is indicated with arrows. (B) qPCR analysis of the orientations of the seven invertible DNA sequences in rough (R) and smooth (S) colony isolates. Data are expressed as the percentage of the population with the sequence in the orientation present in the reference genome (FN545816). Symbols indicate values from individual isolates. (C) qRT-PCR analysis for expression of *cmrR* and *cmrT* in rough and smooth isolates grown on BHIS agar. Data from four biological replicates was analyzed using the ΔΔCt method with *rpoC* as the reference gene and was normalized to the smooth condition. Shown are means with standard deviations. *p < 0.0001, Holm-Sidak multiple *t* test. (D-F) *C*. *difficile* R20291, a TFP-null (*pilB1*) mutant, and an aflagellate (*sigD*) mutant containing the indicated plasmids for manipulation of intracellular c-di-GMP were grown on BHIS agar with or without inducer (2 μg/mL nisin). Shown are images representative of the most common morphology yielded by each strain. Values in each panel indicate the percentage of rough colonies of ≥100 total colonies from at least two independent experiments. All images were taken at ×2 magnification. Data can be found in supplemental file S1 Data. BHIS, brain heart infusion plus yeast; c-di-GMP, cyclic diguanosine monophosphate; *cmr*, colony morphology regulators; DccA, diguanylate cyclase A; *erm*, erythromycin resistance cassette; mut, mutant; R, rough; S, smooth; qPCR, quantitative PCR; qRT-PCR, quantitative real-time PCR; *sigD*, sigma factor D gene; TFP, type IV pilus.

Because colony morphology development exhibited characteristics suggestive of phase variation, we sought to identify the underlying mechanism. To date, the only known phase variation mechanism in *C*. *difficile* involves site-specific DNA recombination resulting in a reversible DNA inversion that is known or predicted to impact gene expression in cis [28]. We postulated that one of the seven known invertible sequences in R20291 regulates the expression of genes involved in colony morphology development, which would be evident as a correlation between the orientation of the invertible sequence and colony morphology (Fig 3A). To test this idea, we differentiated rough and smooth populations and analyzed the orientation of the switches in these isolates using quantitative PCR (qPCR) with orientation-specific primers. For six of the switches, no correlation between morphology and sequence orientation was observed (Fig 3B). However, the orientation of the invertible element Cdi6, which is upstream of the operon CDR20291_3128–3126, showed a strong correlation with colony morphology [28,31]. Each of four independently isolated rough populations contained the sequence predominantly in the orientation previously suggested to favor gene expression, whereas each of the smooth populations contained the sequence predominantly in the inverse orientation [28]. CDR20291_3128–3126 encodes a putative phosphorelay system consisting of two predicted response regulators and a predicted histidine kinase [28]. Accordingly, we named the operon colony morphology regulators RST, where *cmrR* and *cmrT* encode the response regulators and *cmrS* encodes the histidine kinase, and refer to the Cdi6 invertible element as the “*cmr* switch.”

Previous work showed that expression of the *cmrRST* locus is heterogeneous in a population of *C*. *difficile* R20291, occurring in an ON/OFF manner consistent with phase variation [28]. We therefore used quantitative real-time PCR (qRT-PCR) to examine the impact of the orientation of the *cmr* switch on downstream gene expression in rough and smooth isolates. Expression of *cmrR* and *cmrT* was significantly increased in rough isolates relative to smooth (Fig 3C). Combined with the results of orientation-specific PCR of these isolates, these results indicate that bacteria that yield rough colonies bear the *cmr* switch in the ON orientation, whereas the bacteria yielding smooth colonies contain the switch in the OFF orientation. The *cmrRST* locus and its upstream regulatory region containing the *cmr* switch are present in all 65 complete *C*. *difficile* genomes available on the National Center for Biotechnology Information (NCBI) database, with >96% identity at the nucleotide level. This high conservation across diverse ribotypes indicates that this regulatory system, as well as the ability to control its production through phase variation, is important for *C*. *difficile* fitness.

A c-di-GMP binding riboswitch (Cdi-2-2) lies 5′ of *cmrRST* and the invertible sequence and positively regulates expression of the operon (Fig 3A) [40,42]. Additionally, rough and smooth colony morphotypes exhibited inverse swimming and surface motility phenotypes, which is characteristic of c-di-GMP regulation [37,44]. Therefore, we hypothesized that c-di-GMP regulates *C*. *difficile* colony morphology. Using a previously described strategy, intracellular c-di-GMP was increased or decreased by ectopically expressing genes encoding diguanylate cyclase A (DccA) or the EAL phosphodiesterase domain from PdcA, respectively [34,37]. Expression of *dccA* led to the uniform development of rough colonies, whereas the expression of a catalytically inactive DccA (DccA^mut^) did not, indicating that increased c-di-GMP stimulates rough colony formation (Fig 3D). Reducing c-di-GMP through overproduction of the EAL domain did not impact colony morphology, possibly because basal c-di-GMP levels are already low [34]. To determine whether the rough colony effect results from c-di-GMP-mediated inhibition of flagellar motility or activation of TFP-dependent surface motility, *dccA* was expressed in TFP-null *(pilB1*::*erm*) or aflagellate (*sigD*::*erm*) backgrounds. The *pilB1* and *sigD* mutants remained capable of forming both smooth and rough colonies in unmodified and increased c-di-GMP conditions (Fig 3E and 3F). Together, these results indicate dual regulation of the *cmrRST* locus by both phase variation and c-di-GMP. Furthermore, c-di-GMP regulates colony morphology through a mechanism independent of TFP and flagella. To distinguish the spreading colonies developed by rough isolate bacteria from the surface motility imparted by TFP, we refer to the CmrRST-mediated phenomenon as “surface migration.”

### The response regulators CmrR and CmrT regulate colony morphology

Because activation of *cmrRST* expression, whether by increased c-di-GMP and/or inversion of the switch to the ON orientation, promotes the development of rough colonies, we examined the contributions of the response regulators CmrR and CmrT to this process. The *cmrR* and *cmrT* genes were individually expressed under the control of an anhydrotetracycline (ATc)-inducible promoter in *C*. *difficile* R20291 during growth on brain heart infusion plus yeast (BHIS) agar. Both CmrR and CmrT stimulated rough colony formation compared with noninduced controls (S2 Fig). Expression of *cmrT* resulted in a rough phenotype at lower levels of induction than *cmrR*. Interestingly, some levels of induction of *cmrT* inhibited growth, suggesting that *cmrT* expression is toxic. Growth inhibition by CmrT, but not CmrR, was observed in broth culture as well (S1A and S1B Fig).

Response regulators typically have a conserved aspartic acid residue in the phosphoreceiver domain that, when phosphorylated, leads to activation [48]. Mutation of this residue to a glutamic acid often mimics phosphorylation [49,50]. Accordingly, compared with *cmrR* expression, lower levels of induction of *cmrR*^D52E^ expression were required for formation of rough colonies (0.5 ng/mL versus 4 ng/mL ATc, respectively; S2 Fig). Instead of a conserved aspartic acid residue, CmrT contains a glutamic acid at the expected phosphorylation site, precluding the need for a phosphomimic substitution and potentially explaining the lower level of inducer needed to yield rough colonies compared with *cmrR*. Substitution of the phosphorylation site of CmrT with alanine (CmrT-D53A) increased the concentration of inducer needed to obtain rough colonies. However, for the WT and CmrR-D52A mutant, comparable levels of inducer (4 ng/mL ATc) resulted in rough colonies; this may be due to the need for an activating signal that is absent in these assay conditions. That the inactivating mutations did not abolish CmrR and CmrT function suggests that phosphorylation is not required for the activity of these response regulators if they are expressed at high levels.

**Fig 4:**
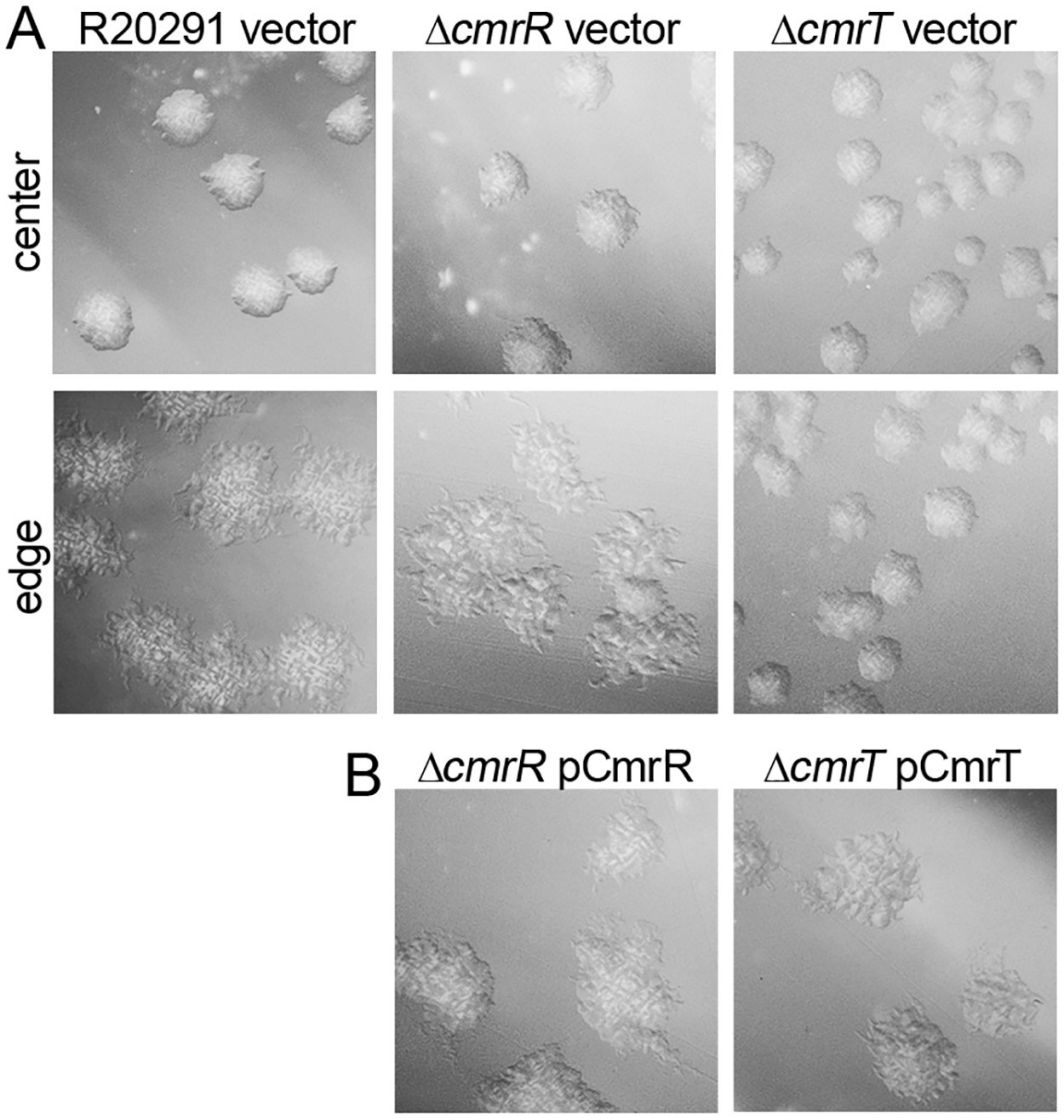
CmrT is required for rough colony formation. (A) Colony morphology of *C*. *difficile* R20291, Δ*cmrR*, and Δ*cmrT* with vector after 24 hours on BHIS-1.8% agar. (B) Complementation of Δ*cmrR* and Δ*cmrT* mutants with expression of the respective genes in trans induced with 2 ng/mL ATc, imaged after 24 hours. All images were taken at ×2 magnification. Representative images from at least two independent experiments are shown. ATc, anhydrotetracycline; BHIS, brain heart infusion plus yeast; *cmr*, colony morphology regulators.

Because CmrR and CmrT promoted the rough colony morphotype, we examined the requirement of *cmrR* and *cmrT* in rough colony formation. Individual in-frame deletions of *cmrR* and *cmrT* were generated in *C*. *difficile* R20291. The mutants and undifferentiated WT R20291 parent were grown on BHIS agar to allow selection of rough colony isolates from the edges (as in Fig 2). Whereas the parent strain and *cmrR* mutant formed both rough and smooth colonies, the *cmrT* mutant did not form rough colonies under the given conditions (Fig 4A). Expression of *cmrT* in trans complemented the *cmrT* mutation, restoring the capacity to form rough colonies (Fig 4B). Importantly, *cmrR* and *cmrT* mutants do not differ in growth rate from WT (S1C Fig). Thus, whereas CmrR and CmrT both impact colony morphology development when overproduced, only CmrT is required for rough colony formation under the tested conditions.

### CmrR and CmrT inversely regulate swimming motility and surface migration

**Fig 5:**
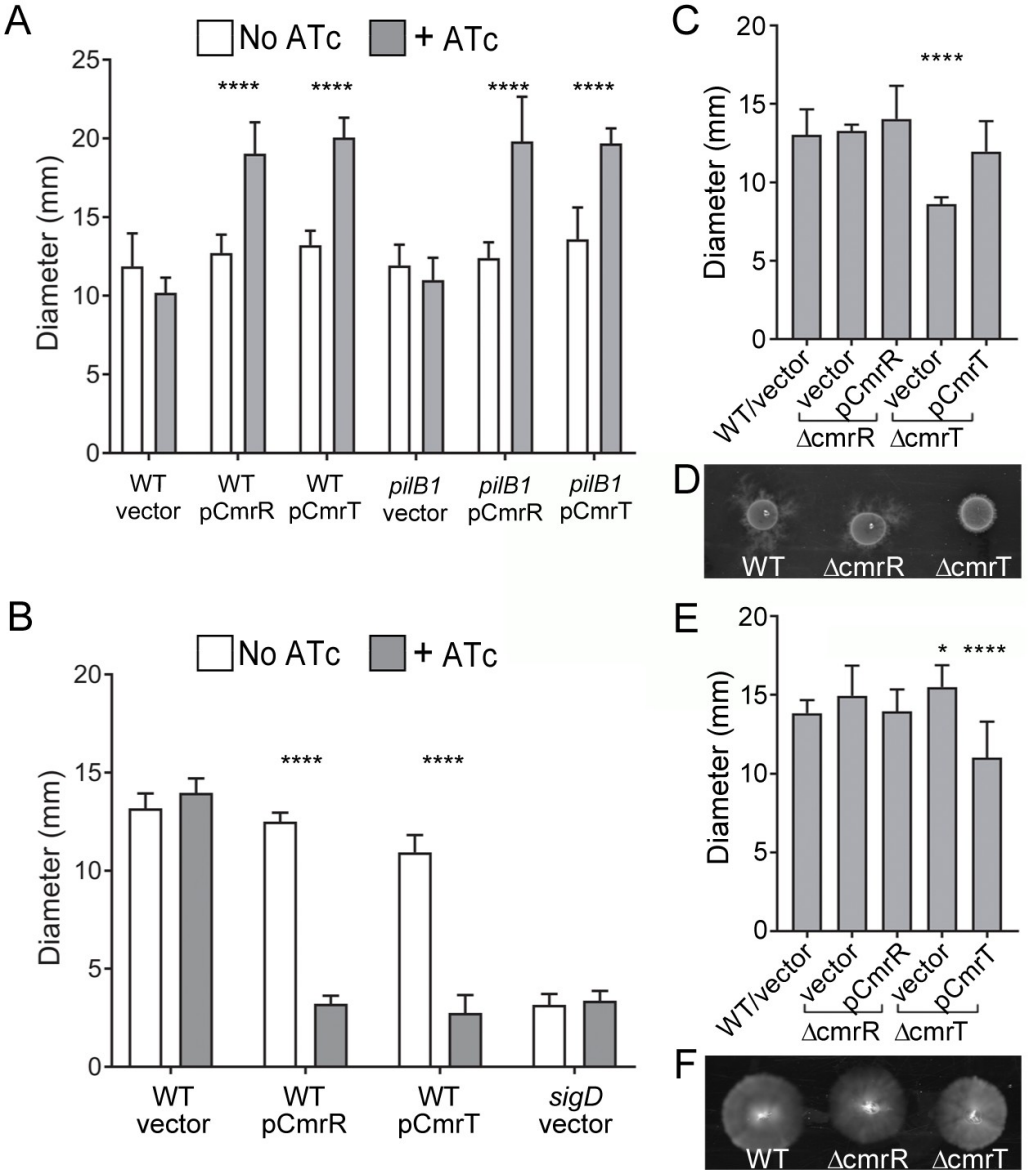
CmrR and CmrT inversely regulate surface and swimming motility. *C*. *difficile* strains were assayed for surface motility on BHIS-1.8% agar 1% glucose after 72 hours (A-C) and for swimming motility through 0.5× BHIS-0.3% agar after 48 hours (D-F). (A) *C*. *difficile* R20291 (WT) and a TFP-null (*pilB1*) mutant, each with plasmids for expression of *cmrR*, *cmrT*, or a vector control, assayed for surface motility with or without ATc (10 ng/μL for pCmrR, 2 ng/μL for pCmrT). (B) Surface motility of WT, Δ*cmrR*, and Δ*cmrT* strains with vector or respective expression plasmids for complementation. (C) Representative image of surface motility. (D) *C*. *difficile* R20291 (WT) and a nonmotile *sigD* mutant with pCmrR, pCmrT, or vector as indicated, assayed for swimming motility with or without ATc (10 ng/μL for pCmrR, 2 ng/μL for pCmrT). (E) Swimming motility of WT, Δ*cmrR*, and Δ*cmrT* strains with vector or complementation plasmids in the presence of 0.2 ng/μL. A nonmotile *sigD* mutant was included as a control. (F) Representative image of swimming motility. Shown are the means and standard deviations of the diameters of motility growth. *p < 0.05, ****p < 0.0001, two-way ANOVA and Tukey’s posttest comparing +/− ATc (A, D) or one-way ANOVA with Dunnett’s posttest comparing values to WT/vector (B, E). Data can be found in supplemental file S1 Data. ATc, anhydrotetracycline; BHIS, brain heart infusion plus yeast; *cmr*, colony morphology regulators; *sigD*, sigma factor D gene; TFP, type IV pilus; WT, wild-type.

Because bacteria isolated from rough and smooth colonies differed in surface migration and swimming motility (Fig 2), we assessed the roles of CmrR and CmrT in these processes. Ectopic expression of *cmrR* or *cmrT* in R20291 significantly increased surface migration compared with uninduced and vector controls (Fig 5A, S3A and S3B Fig). Enhanced surface migration was also observed in a TFP-deficient (*pilB1*::*erm*) background, again indicating that CmrR- and CmrT-mediated surface migration is independent of TFP (Fig 5A, S3A and S3B Fig). Conversely, *cmrR* and *cmrT* expression inhibited swimming motility of R20291 (Fig 5D, S3C and S3D Fig). These data are consistent with the increased surface migration and decreased swimming motility exhibited by rough colony isolates (*cmrRST* ON), which are the inverse of the motility phenotypes of the smooth colony isolates (*cmrRST* OFF). Expression of *cmrR* and *cmrT* alleles with inactivating alanine substitutions resembled the WT allele, suggesting again that high levels of expression overcome the need for phosphorylation (S3 Fig). However, as seen with colony morphology (S2 Fig), the *cmrR-*D52E allele increased surface migration and decreased swimming motility at lower levels of induction relative to the WT allele, suggesting that the amino acid substitution increased activity (S3 Fig). Expression of *cmrR* or *cmrT* did not alter transcript levels of representative flagellum or TFP genes (S4 Fig), suggesting that the observed differences in surface migration and swimming motility occur through posttranscriptional regulation or through an alternative mechanism.

To further define the role of *cmrRST*, the *cmrR* and *cmrT* mutants and relevant controls were evaluated for surface migration and swimming motility. In both assays, the *cmrR* mutant behaved comparably to the R20291 parent (Fig 5B–5F), indicating that CmrR is dispensable for these processes. However, the *cmrT* mutant exhibited significantly reduced surface migration, a defect that was complemented by expressing *cmrT* in trans. The opposite effect was observed for swimming motility; the *cmrT* mutant showed greater motility compared with the parent strain. CmrT thus appears to be the dominant response regulator of the CmrRST system for control of rough colony formation, surface migration, and swimming motility in vitro.

### CmrR and CmrT promote bacterial chaining

**Fig 6:**
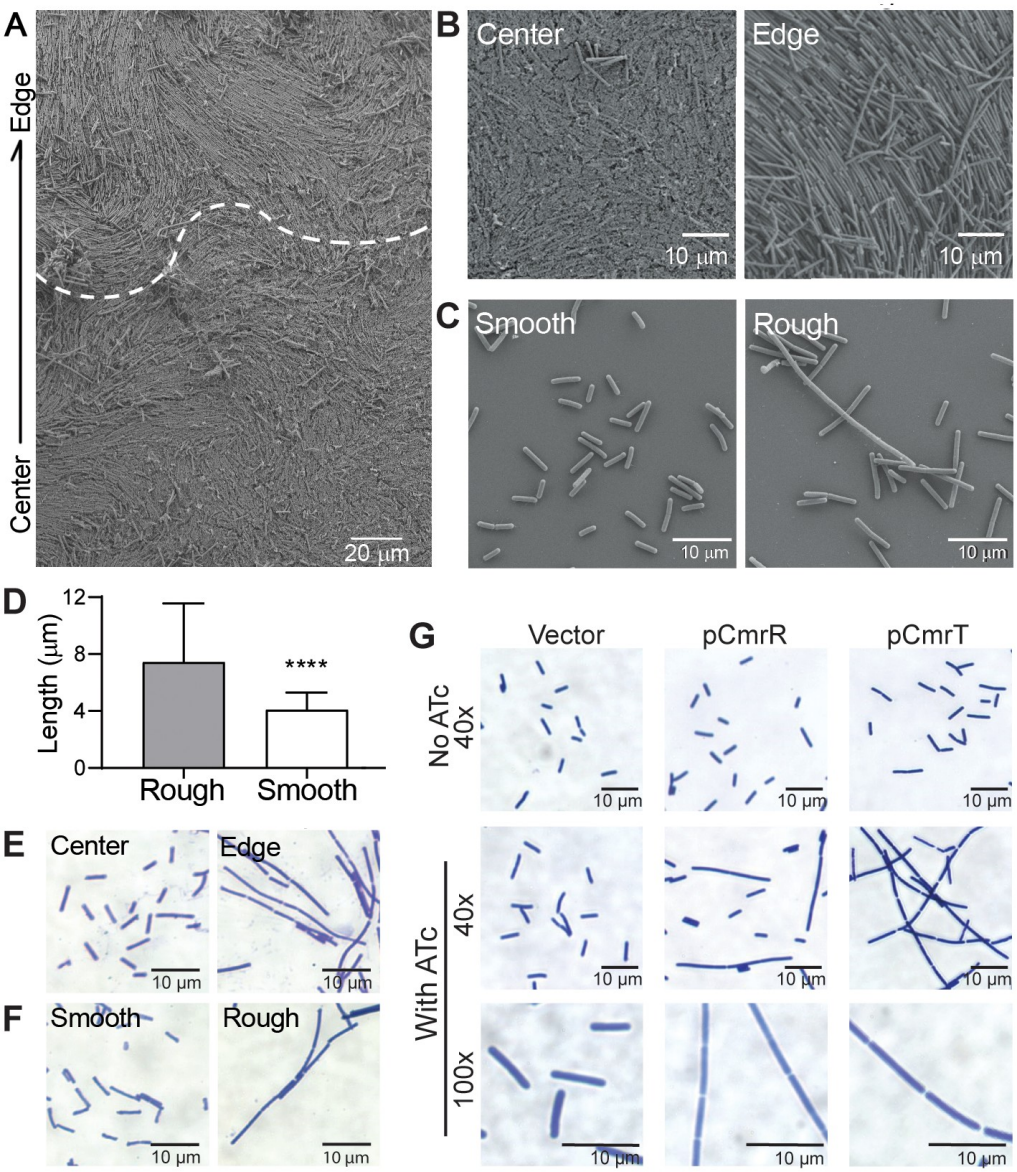
CmrR and CmrT promote bacterial chaining. (A) SEM images of a whole R20291 colony after 3 days of growth on BHIS agar. (A) A colony excised on an agar slice at 1 × 10^3^ magnification. The arrow denotes the orientation of the image with respect to the colony. The dotted line marks the apparent transition from the colony edge and center. (B) Shown are 2.5 × 10^3^ magnification images of cells at the colony center and edge. (C) Shown are 4.5 x 10^3^ magnification images of rough and smooth cell suspensions. Samples were grown in broth culture prior to fixation. Shown are representative images from two biological replicates. (D) Quantification of cell lengths obtained from SEM images using ImageJ. Cells were measured from at least seven images from two biological replicates (rough *n =* 574, smooth *n =* 457). ****p < 0.0001 by unpaired *t* test. Data can be found in supplemental file S1 Data. (E, F) Gram stain of *C*. *difficile* samples taken directly from the center and edge of a colony (E) or from rough and smooth colonies (F) at ×60 magnification. G) Gram stain of *C*. *difficile* with plasmids for expression of *cmrR* or *cmrT*, or a vector control were grown on BHIS agar with and without inducer (10 ng/mL ATc for vector and pCmrR; 2 ng/mL ATc for pCmrT). Magnifications are indicated. Shown are representative images from two independent experiments. ATc, anhydrotetracycline; BHIS, brain heart infusion plus yeast; *cmr*, colony morphology regulator*s*; SEM, scanning electron microscopy.

Phenotypic analysis of motility and quantification of flagellum and TFP transcripts indicate that CmrRST-mediated colony morphology occurs independently of these surface structures. Cell morphology has also been shown to affect gross colony morphology [26,51,52], so we investigated whether cell morphology differs between rough and smooth morphotypes. Examination of *C*. *difficile* colonies by scanning electron microscopy (SEM) revealed the presence of the expected bacilli toward the center, as well as elongated cells, particularly along the edge of the colony (Fig 6A). These elongated cells appeared as organized bundles corresponding to the tendrils apparent in the macrocolony, suggesting a role for the elongated bacteria in expansion of rough colonies. Consistent with this, SEM of bacteria derived from smooth and rough colonies revealed dichotomous cellular morphologies. Smooth colony–derived bacteria appeared as standard bacilli (4.089 ± 1.207 μm in length), whereas rough colony–derived cells were longer (7.428 ± 4.130 μm) and sometimes exhibited an extremely elongated shape, resembling a bacterial filament or chain (Fig 6B and 6C). To determine whether the elongated cells result from filamentation or cell chaining, cells from rough and smooth colonies were Gram stained. Whereas cells from smooth colonies consisted of the expected single or double rods, cells from rough colonies more commonly appeared in bacterial chains of three or more cells (Fig 6E and 6F). Septa separating cells within chains were clearly visible, differentiating this morphology from filamentation. Differences in processing may explain why cell chains were more common in Gram-stained samples than in SEM-imaged samples; processing of samples for SEM may have disrupted chains.

Because *cmrRST* expression favors rough colony development, we examined the roles of CmrR and CmrT in the chaining phenotype. In contrast to uninduced and vector controls, bacteria overexpressing *cmrR* or *cmrT* also displayed cell chaining (Fig 6G). Based on our surface motility data, we predicted that though CmrR and CmrT both contribute to cell chaining and elongation, only the Δ*cmrT* mutant would be deficient in these phenotypes. We imaged Gram-stained WT, Δ*cmrR*, and Δ*cmrT* cells collected from the edges of motile spots (S6A and S6B Fig). The Δ*cmrR* mutant displayed a slight decrease in cell length as compared with the WT control but still appeared as elongated cells and cell chains. However, Δ*cmrT* cells were significantly shorter than WT and appeared only as single or double cells. Together, these results indicate that rough colonies contain bacterial chains whose formation is promoted by CmrRST.

To better understand the contribution of cell chaining to the expansion of a colony on a surface, we visualized WT, Δ*cmrR*, and Δ*cmrT* grown on agar. WT, Δ*cmrR*, and TFP-deficient strains displayed elongated cells in bundled cell chains, and these structures appeared to expand in an ordered manner away from the colony edge (S5C Fig). However, the Δ*cmrT* strain, which is defective in surface motility, did not display these long cell bundles. Together, these data suggest that CmrT-mediated changes in cell morphology and chaining contribute to the movement of *C*. *difficile* across a surface.

Bacterial motility, cell morphology, and adherent behaviors are central to biofilm development, and chaining and filamentation can also affect surface adhesion and biofilm formation [53–56]. We therefore examined the role of CmrRST in *C*. *difficile* biofilm formation. Bacteria isolated from rough and smooth colonies and the *cmrR* and *cmrT* mutants were assayed for biofilm production in rich medium on plastic as described previously [37,38]. The *cmrR* mutant showed significantly increased biofilm formation compared with the rough isolate, whereas the *cmrT* mutant showed no significant difference (S6 Fig), suggesting that CmrR negatively regulates biofilm formation.

### Phase-variable colony morphology via CmrRST impacts *C*. *difficile* virulence

**Fig 7:**
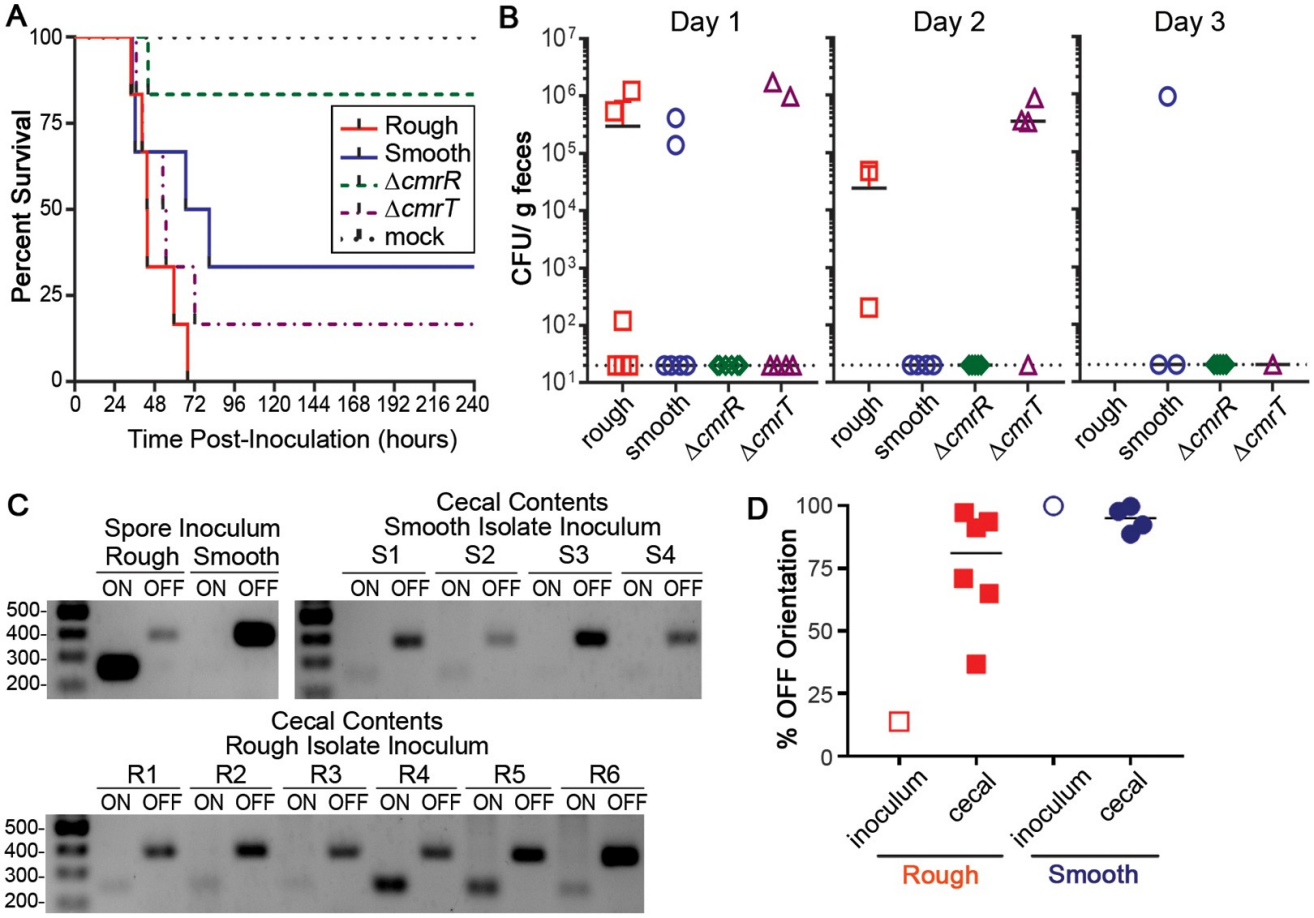
Colony morphology and CmrR impact *C*. *difficile* virulence. Male and female Syrian golden hamsters were inoculated with 5,000 spores of the indicated *C*. *difficile* strains/isolates. (A) Kaplan-Meier survival data showing time of morbidity and euthanasia. Log-rank test: rough versus Δ*cmrR*, *p* = 0.003; smooth versus Δ*cmrR*, *p* = 0.098, log-rank test. (B) CFU enumerated in feces collected at 24-hour intervals postinoculation. Symbols indicate CFU from individual animals, and bars indicate the means. (C) Orientation of the *cmr* switch determined by PCR for the spore inocula and cecal contents of moribund hamsters (rough R1–6, smooth S1–4) with detectable *C*. *difficile*. (D) Quantification of *cmr* switch orientation by qPCR in spore inocula and cecal contents of moribund hamsters. Data are expressed as the percentage of the population with the sequence in the OFF orientation. Symbols indicate values from individual hamsters, and horizontal bars indicate the median. Values of rough and smooth cecal contents do not differ significantly from each other (*p >* 0.44, one-way ANOVA). Data can be found in supplemental file S1 Data. CFU, colony-forming unit; *cmr*, colony morphology regulators; qPCR, quantitative PCR; R, rough; S, smooth.

Given the broad role of CmrRST in controlling *C*. *difficile* physiology and behaviors, we predicted that the *cmrR* or *cmrT* mutants would have altered virulence in a hamster model of *C*. *difficile* infection. Male and female Syrian golden hamsters were inoculated with spores of Δ*cmrR* or Δ*cmrT*, or smooth or rough isolates of R20291, which remain capable of phase-varying *cmrRST* expression. Notably, the strains tested did not differ in sporulation or germination rates in vitro (S7 Fig). The animals were monitored for disease symptoms and euthanized when moribund as described in the Materials and methods. Hamsters infected with Δ*cmrR* showed increased survival compared with those infected with the rough isolate of the R20291 parent (*p* = 0.003, log-rank test). Survival of hamsters infected with Δ*cmrR* was also greater than that of animals infected with the smooth isolate, but the difference did not reach statistical significance (*p* = 0.098, log-rank test). Whereas the mean times to morbidity were comparable for the animals that did succumb (44.08 hours for Δ*cmrR*, 48.15 ± 12.87 hours for rough, 54.69 ± 22.90 hours for smooth), most of the animals inoculated with Δ*cmrR* survived (Fig 7A). For animals infected with Δ*cmrT*, both time to morbidity and percent animal survival were equivalent to those infected with the rough and smooth isolates.

To evaluate intestinal colonization, fecal samples were collected every 24 hours postinoculation, and dilutions were plated on taurocholate cycloserine cefoxtin fructose agar (TCCFA) medium (containing the spore germinant taurocholic acid) to enumerate *C*. *difficile* colony-forming units (CFU). All animals infected with Δ*cmrT*, rough, and smooth isolates yielded detectable CFU at the time of collection preceding disease development and euthanasia (Fig 7B). However, we observed no correlation between bacterial load and disease onset or severity. The number of Δ*cmrR* CFU was never above the limit of detection in feces of infected animals (Fig 7B). These data indicate that CmrR, but not CmrT, is important for the ability of *C*. *difficile* to colonize the intestinal tract and cause disease in the hamster model.

To determine whether the attenuation of the Δ*cmrR* mutant was due to reduced toxin levels, we evaluated the effect of CmrR and CmrT on TcdA production. Western blot analysis showed that Δ*cmrR* does not produce significantly lower levels of TcdA toxin than rough and smooth isolates in vitro, suggesting that the virulence defect of Δ*cmrR* occurs through a toxin-independent mechanism (Fig 7C and 7E). Interestingly, Δ*cmrT* produced slightly higher TcdA levels by western blot. However, overexpression of *cmrR* and *cmrT* in R20291 did not significantly alter *tcdA* transcript abundance, suggesting that CmrRST does not transcriptionally regulate toxin gene expression.

Because the Δ*cmrR* strain was attenuated in hamsters, we expected the smooth isolate (CmrRST OFF) to be attenuated compared with the rough isolate (CmrRST ON). Whereas hamsters infected with the rough isolate all succumbed to disease, 33% of hamsters inoculated with the smooth isolate survived, suggesting moderately lower virulence of the smooth isolate (Fig 7A). However, this difference did not reach statistical significance, because the survival times of hamsters that became moribund were not significantly different (*p* = 0.112, log-rank test), and both isolates were able to colonize (Fig 7A and 7B).

We considered the possibility that the rough and smooth isolates, though chosen based on distinct colony morphologies, are WT and capable of phase variation. Accordingly, selective pressures in the intestinal tract may have resulted in a phenotypic switch during infection, mitigating the observed differences in virulence. To test this, we determined the orientations of the *cmrRST* switch in the spore preparations and the contents of the cecums collected from infected animals immediately after sacrifice. DNA obtained from cecal contents was subjected to PCR with two primer sets that amplify from the *cmrRST* switch in a particular orientation. Analysis of the *cmrRST* switch orientation in the spore inocula indicate that the rough isolate consisted of bacteria with the switch primarily in the ON orientation, whereas the smooth isolate contained those primarily in the OFF orientation, consistent with prior in vitro analyses (Fig 7C and 7D). In the cecal contents of hamsters inoculated with the smooth isolate, *C*. *difficile* retained the *cmrRST* switch predominantly in the OFF orientation (94.56% ± 2.52% OFF; Fig 7D). In contrast, cecal contents from rough isolate–infected animals also contained the switch in a mixed or predominantly OFF orientation (75.71% ± 9.44% OFF), indicating that the rough (ON) isolate inoculum underwent phase variation during infection. These data suggest that either ON/OFF switch rates are altered in favor of the OFF orientation during acute, short-term infection, and/or there is a selective pressure for the *cmrRST*-OFF state and corresponding phenotypes.

## Discussion

Phase variation provides a mechanism for generating phenotypic heterogeneity in bacterial populations to enhance the survival of the population as a whole when encountering detrimental environmental stresses. In this study, we characterized the phase variation of a signal transduction system, CmrRST, which we found broadly impacts *C*. *difficile* physiology and behavior. During growth in vitro, *C*. *difficile* populations consist of bacterial subpopulations that are CmrRST ON, yielding rough colonies, and CmrRST OFF, yielding smooth colonies. These subtypes display divergent motility phenotypes in vitro, with smooth colony isolates showing increased swimming motility and rough colony isolates displaying enhanced surface motility. The identification of growth conditions that segregate for the respective morphological variants, each with enhanced motility in the corresponding growth condition, indicates a selective advantage for that motility behavior.

Although ectopic expression of either response regulator, CmrR or CmrT, increased surface motility, only CmrT is required for surface migration and rough colony development, suggesting that CmrT is the dominant regulator under these conditions. Alternatively, CmrR may also play a role but requires an activating signal through the histidine kinase CmrS to be functional. The latter possibility is supported by the observation that a phosphomimic substitution at the predicted phosphorylation site in the receiver domain increases CmrR activity. In contrast, because CmrT contains a glutamic acid at this site, it likely does not require activation by a histidine kinase and instead acts constitutively. For this reason, the receiver domain of CmrT may be considered a pseudoreceiver domain [48,57].

In the hamster model of acute infection, the *cmrR* mutant did not detectably colonize and exhibited reduced virulence, indicating a role for this signal transduction system during infection. In contrast with the in vitro motility phenotypes driven primarily by CmrT, the CmrR regulator appears to be critical for virulence. The divergent roles for the regulators suggest that an activating signal for CmrR is present in the host intestine, though it remains possible that CmrT activity is somehow inhibited during infection. A similar mechanistic distinction between the regulators may explain the role of CmrR, but not CmrT, in biofilm formation in vitro. Whether CmrR and CmrT control distinct regulons and act cooperatively and/or antagonistically is unknown and currently under investigation.

Compared with the strong attenuated virulence of the *cmrR* mutant, the rough and smooth isolates exhibited modest differences in virulence. The latter result may have arisen from the ability of the isolates to phase vary, resulting in convergent phenotypes due to selective pressures in the intestinal environment. Tracking of the *cmr* switch orientations in the inocula and cecal contents at the experimental end point indicate that the rough variant (*cmrRST* ON) switched to populations with the *cmr* switch in a mixed or predominantly OFF orientation. That phase variation of CmrRST occurred during infection may have resulted in diminished differences in virulence between the rough and smooth variants. In addition, these findings imply the presence of a selective pressure that favors the *cmrRST-*OFF phase variant, at least in the later stages of infection. However, the high conservation of the *cmrRST* locus and the upstream regulatory region indicate that this regulatory system and its ability to phase vary confer a fitness advantage. The colonization defect of the *cmrR* mutant suggests that signaling through CmrR contributes to initial colonization, and therefore, the ability to generate a CmrRST-ON subpopulation may be important at an early stage of infection. In our study, cecal contents were harvested at the experimental end point when disease was fulminant, and the collection of samples after more-frequent intervals is limited by the short duration of the infection and low bacterial load prior to 24 hours postinoculation. This sampling strategy may not capture transient changes in the *cmr* switch bias during different stages of infection, over a longer infection, or in a different location in the intestinal tract. Testing the impact of *cmrR* and *cmrT* mutations in a phase-locked ON background as well as a double *cmrR cmrT* mutant may better reveal the role of this system in *C*. *difficile* virulence.

Unlike phase variation mechanisms that control the production of a specific surface factor, the phase variation of the CmrRST system is poised to coordinate the expression of multiple pathways. Previous studies have described phase variation of proteins with regulatory function resulting in broad changes in gene expression. A few transcriptional regulators have been reported to phase vary, including the global virulence gene regulators BvgAS in *Bordetella* and the response regulator FlgR in *Campylobacter jejuni* [58–60]. In *C*. *difficile*, phase variation of flagellar genes impacts the transcription of the alternative sigma factor gene *sigD*, which couples the expression of flagellum and toxin genes, linking virulence factor production to the orientation of the flagellar switch [19,61]. Coordinated regulation of genes resulting in global changes in transcription can also result from phase variation of DNA modification systems, as observed in *Haemophilus influenza*, *Helicobacter pylori*, *Salmonella enterica*, and *Streptococcus* strains [62–67]. In *S*. *pneumoniae*, for example, DNA inversions within three genes encoding the sequence recognition factor of a type I restriction-modification (RM) system allow for changes in genomic methylation patterns, resulting in phase-variable regulation of virulence factors including capsule [67,68]. The CmrR and CmrT response regulators each contain DNA binding domains and have the capacity to regulate gene transcription. Therefore, inversion of the *cmr* switch likely also indirectly controls the expression of multiple genes in a phase-variable manner.

The CmrRST system regulates multiple processes, but the basis for the phenotypic changes are unclear. Colony morphology and motility assays indicate TFP- and flagellum-independent mechanisms, and the transcription of TFP and flagellum genes is not affected by CmrR and CmrT. TFP-independent surface migration has not previously been described in *C*. *difficile*. Examination of cellular morphology indicates that CmrRST promotes bacterial chaining. *Bacillus subtilis* also exhibits cell chaining, and this chaining may contribute to bacterial migration as cells are pushed along the axis of growth and division [69–72]. In support of this hypothesis, the edges of *C*. *difficile* motile spots exhibit well-organized bundled chains of elongated cells that extend away from the center and are absent without *cmrT*. This mechanism appears similar to gliding motility in *Clostridium perfringens*, although that process requires TFP [73,74]. Bacterial chaining is observed in a number of bacterial species and can affect flagellar motility, surface adhesion, biofilm formation, susceptibility to phagocytosis, and virulence [52–56,69,75–78]. Chaining of *C*. *difficile* through regulation by CmrRST may similarly alter motility behaviors and persistence in the intestine. How CmrRST mediates changes to cell chaining, colony morphology, and motility is unknown, and ongoing work to define the CmrRST regulon may reveal the underlying mechanisms.

The complexity of *cmrRST* regulation not only alludes to the importance of controlling CmrRST activity but also highlights the growing link between phase variation and c-di-GMP signaling in *C*. *difficile*. Expression of flagellar genes is also modulated by both a c-di-GMP binding riboswitch and an invertible element [19,41,44], as well as two additional phase-variable genes in *C*. *difficile* that encode c-di-GMP phosphodiesterases [28]. In the case of flagellar gene regulation, c-di-GMP inhibits transcription through a riboswitch that induces transcription termination, and the invertible flagellar switch further controls expression through an uncharacterized posttranscriptional mechanism. For *cmrRST* expression, c-di-GMP positively regulates transcription, and the *cmr* switch controls phase variation through an undefined mechanism [28,40]. The hierarchy between these regulatory elements is unclear. A σ^A^-dependent promoter lies 5′ of both the riboswitch and *cmr* switch [42]. The invertible sequence may contain an additional promoter to drive expression when properly oriented [4,79]. Alternatively, the upstream σ^A^-dependent promoter may be the only site of transcriptional initiation, and the invertible element may contain another regulatory sequence that modulates expression [80].

Finally, this and other recent work underscore that genetically clonal *C*. *difficile* strains, as well as isogenic mutants, may differ phenotypically because of phase variation. The seven sites of site-specific DNA recombination in the *C*. *difficile* genome can be inverted independently, and this study shows that in vitro culturing conditions can change the biases of switch orientations in the overall population. From a research standpoint, this can introduce significant variability into otherwise controlled experiments. This concern may be especially significant in infection studies, in which differences in switch biases in inocula may skew apparent outcomes. Caution should be taken in interpreting such data, as observed differences may be due to differences in expression of phase-variable traits rather than the targeted mutations.

Through phase variation, populations of bacterial pathogens can rapidly adapt to changing environmental pressures, balancing the need to move, adhere, and avoid immune recognition through the course of infection. The phase variation of CmrRST may allow alternating modes of surface or swimming motility in *C*. *difficile* as needed during infection—swimming motility (*cmrRST* OFF) may allow exploration and colonization of new sites, whereas bacterial chaining (*cmrRST* ON) may allow the spread of bacteria along the epithelial surface once contact has been made. Further work illustrating the role of CmrRST will provide important insights into *C*. *difficile* pathogenesis.

## Materials and methods

### Ethics statement

All animal experimentation was performed under the guidance of veterinarians and trained animal technicians within the Emory University Division of Animal Resources (DAR). Animal experiments were performed with prior approval (approval number 201700396) from the Emory University Institutional Animal Care and Use Committee (IACUC) under protocol #DAR-2001737-052415BA. Animals considered moribund were euthanized by CO_2_ asphyxiation followed by thoracotomy in accordance with the Panel on Euthanasia of the American Veterinary Medical Association. The University is in compliance with state and federal animal welfare acts and the standards and policies of the Public Health Service, including documents entitled "Guide for the Care and Use of Laboratory Animals" (National Academy Press, 2011), "Public Health Service Policy on Humane Care and Use of Laboratory Animals" (September 1986), and Public Law 89544 with subsequent amendments. Emory University is registered with the United States Department of Agriculture (57-R-003) and has filed an assurance of compliance statement with the Office of Laboratory Animal Welfare of the National Institutes of Health (A3180–01).

### Growth and maintenance of bacterial strains

*C*. *difficile* strains were maintained in an anaerobic environment of 85% N_2_, 5% CO_2_, and 10% H_2_. *C*. *difficile* strains were cultured in Tryptone Yeast (TY; 30 g/L Bacto tryptone, 20 g/L yeast extract, 1 g/L thioglycolate) broth or BHIS (37 g/L Bacto brain heart infusion, 5 g/L yeast extract) media at 37 °C with static growth. *E*. *coli* strains were grown in Luria Bertani medium at 37 °C with aeration. Antibiotics were used where indicated at the following concentrations: chloramphenicol (Cm), 10 μg/mL; thiamphenicol (Tm), 10 μg/mL; kanamycin (Kan), 100 μg/mL; ampicillin (Amp), 100 μg/mL. S1 Table lists descriptions of the strains and plasmids used in this study.

### Differentiation of rough and smooth colonies

To differentiate rough and smooth colonies from WT *C*. *difficile* strains, 5 μL of liquid cultures were spotted onto BHIS agar plates and grown for 48–72 hours. For rough and smooth colonies, all growth was collected and plated in dilutions on BHIS agar. For predominantly rough colonies, growth was collected from the filamentous edges [18,34]. For predominantly smooth colonies, growth was collected from the center of the spot (our stock of *C*. *difficile* R20291 consists primarily of bacteria yielding smooth colonies, so the spot center represents the inoculum). For enumeration of rough and smooth colonies, serial dilutions were plated, rough and total CFU were counted, and data were expressed as percent rough CFU.

### Microscopy

For whole-colony imaging, colonies were grown on BHIS plates for 24 hours prior to imaging. For strains containing plasmids, the BHIS agar medium contained 10 μg/mL Tm. For strains carrying pDccA, pDccA^mut^, pEAL, or pMC-Pcpr, 2 μg/mL nisin was included to induce gene expression. For strains with pCmrR, pCmrT, or mutant derivatives, ATc was added at the indicated concentrations. Colonies were imaged at ×2–8 magnification using a Zeiss Stereo Discovery V8 dissecting microscope with a glass stage and light from the top.

For imaging of motile spots, R20291 WT, Δ*cmrR*, Δ*cmrT*, and *pilB1-*null strains were grown overnight in BHIS and then spotted on BHIS 1.8% agar 1% glucose. After 72 hours of growth, colonies were images using a Keyence BZ-X810 microscope.

For Gram stain imaging, R20291 WT rough or smooth, Δ*cmrR*, or Δ*cmrT* colonies were differentiated as previously described and heat fixed on a glass slide. Cells were Gram stained (BD Kit 212524) and visualized at ×40–80 magnification using an Olympus BX60 compound microscope or a Keyence BZ-X810 microscope. For quantification of cell length, at least two images from two biological replicates were analyzed using ImageJ

For visualization by SEM, R20291 rough and smooth isolates and the *sigD* mutant were grown overnight in TY broth and washed once with phosphate buffered saline (PBS). For visualization of colony structure, R20291 was spotted on BHIS agar and grown for 3 days. The colony was excised on agar and fixed. All samples were fixed in 4% glutaraldehyde in 150 mM sodium phosphate buffer. The samples were dehydrated through increasing concentrations of ethanol and dried using carbon dioxide as the transition solvent in a Samdri-795 critical-point dryer (Tousimis Research Corporation, Rockville, MD). Coverslips were mounted on aluminum stubs with carbon adhesive tabs, followed by a 5-nm thickness platinum sputter in a Hummer X sputter coater (Anatech USA, Union City, CA). Images were taken with a working distance of 5 mm and a 130-μm aperture using a Zeiss Supra 25 field emission SEM (FESEM) operating at 5 kV (Carl Zeiss SMT, Peabody, MA). For quantification of cell length, at least seven images from two biological replicates were analyzed using ImageJ.

### qPCR analysis of invertible element orientations

qPCR was used to quantify the percent of the population with each of the seven invertible elements in a given orientation as described previously [28]. Rough and smooth colonies were collected from a BHIS plate, and genomic DNA was purified. qPCR was performed with SensiMix SYBR (BioLine). Twenty-microliter reactions with 100 ng of genomic DNA and 100 nM primers were used. Reactions were run on Lightcycler 96 system (Roche) with the following three-step cycling conditions: 98 °C for 2 minutes, followed by 40 cycles of 98 °C for 30 seconds, 60 °C for 1 minute, and 72 °C for 30 seconds. Quantification was done using the ΔΔCt method, with *rpoA* as the reference gene and the indicated reference condition. All primers used in this and other experiments are listed in S2 Table.

### Construction of Δ*cmrR* and Δ*cmrT* in *C*. *difficile* R20291

Markerless deletions of *cmrR* (locus tag CDR20291_3128) and *cmrT* (locus tag CDR20291_3126) were done using a previously described *codA*-based allelic exchange method with minor modifications [81]. Briefly, genomic fragments of approximately 1,100–1,300 bp were PCR amplified upstream and downstream of *cmrR* and *cmrT* with the following primers sets: OS158 and OS266 (*cmrR*, upstream), OS163 and OS267 (*cmrR*, downstream); OS268 and OS269 (*cmrT*, upstream), OS271 and OS283 (*cmrT*, downstream). Complementary overlapping sequences were added to the 5′ end of primers to allow for accurate fusion of all PCR products into PmeI-linearized pMTL-SC7215 vector using Gibson Assembly Master Mix (New England BioLabs). The resulting plasmids were then conjugated into *C*. *difficile* R20291 strain (GenBank accession: CBE06969.1) using the heat-stimulated conjugation method described elsewhere [82]. Mutants were selected as previously described and screened by colony PCR for the presence of the correct left and right homology-chromosomal junction and the absence of respective *cmrR* and *cmrT* coding sequence [81]. All PCR products were Sanger sequenced to confirm the correct genetic construct and the absence of any secondary mutations.

### Surface and swimming motility assays

For surface motility assays, 5 μL of overnight (16–18 hours) cultures were spotted onto BHIS-1.8% agar supplemented with 1% glucose [39]. For swimming motility assays, 1 μL of overnight cultures was inoculated into 0.5× BHIS-0.3% agar [19]. For plasmid-bearing strains in both assays, the medium was supplemented with 10 μg/mL Tm. Where indicated, ATc was included at the indicated concentrations to induce expression. At 24-hour intervals, the diameters of growth were taken as the average of two perpendicular measurements. For measurements of surface colony diameters, the first measurement corresponds to the widest part of the colony including any tendrils, and the second corresponds to a perpendicular measurement regardless of the presence of a tendril. These values were averaged and used as the colony diameter as described previously [37]. Data from at least eight biologically independent cultures were analyzed using a one-way ANOVA to determine statistical significance. The plates were photographed using a Syngene G:Box imager.

### Biofilm assay

Biofilm assays were done as previously described [38]. Overnight cultures of *C*. *difficile* were diluted 1:100 in BHIS 1% glucose 50 mM sodium phosphate buffer (pH 7.5) in 24-well polystyrene plates. After 24 hours of growth, supernatants were removed, and the biofilms were washed once with PBS and then stained for 30 minutes with 0.1% crystal violet. After 30 minutes, the biofilms were washed again with PBS, and the crystal violet was solubilized with ethanol. Absorbance was read at 570 nm. Four independent experiments were performed.

### RNA isolation and real-time PCR

For qRT-PCR analysis of flagellar gene expression, *C*. *difficile* with vector or *cmrR*/*cmrT* expression plasmids was grown for 48 hours in 0.5× BHIS-0.3% agar to express flagellar genes. Bacteria were recovered and cultured in TY broth with 10 ng/mL ATc for vector and pCmrR and 2 ng/mL ATc for pCmrT. For qRT-PCR analysis of toxin gene expression, *C*. *difficile* with vector or *cmrR*/*cmrT* expression plasmids was grown overnight in TY broth and diluted into BHIS broth with 10 ng/mL ATc for vector and pCmrR and 2 ng/mL ATc for pCmrT. For both experiments, samples were collected at the midexponential phase and preserved in 1:1 ethanol-acetate. For qRT-PCR analysis of *cmrR* and *cmrT* transcript abundance in rough and smooth isolates, rough and smooth colonies were differentiated as described above and streaked on BHIS agar. After 24 hours growth, colonies were collected and preserved in 1:1 ethanol-acetate.

RNA was isolated, treated with DNase I, and reverse transcribed including a no-reverse transcriptase control as previously described [32,34]. Real-time PCRs were done using 2 ng of cDNA, primers at a final concentration of 500 nM, and SYBR Green Real-Time qPCR reagents (Thermo Fisher) with an annealing temperature of 55 °C. Primers used were as follows: R856-R857, *flgB*; R858-R859, *flgM*; R1063-R1064, *fliC*; R930-R931, *pilA1*; R852-R853, *tcdA*; R2298-R2299, *cmrR*; and R2537-R2538, *cmrT*. The data were analyzed using the ΔΔCt method with *rpoC* (R850-R851) as the reference gene.

### Animal experiments

All animal experimentation was performed under the guidance of veterinarians and trained animal technicians within the Emory University DAR. Animal experiments were performed with prior approval from the Emory University IACUC. *C*. *difficile* spores were collected from 70:30 sporulation broth after 3 days of growth [83]. The spores were purified using a sucrose gradient and stored in PBS with 1% bovine serum albumin (BSA) as described previously [45,84]. Prior to inoculation, the spores were enumerated by plating serial dilutions on BHIS agar containing 0.1% sodium taurocholate.

*C*. *difficile* spores were collected from 70:30 sporulation broth after 3 days of growth [83]. The spores were purified using a sucrose gradient and stored in PBS with 1% BSA as described previously [45,84]. Prior to inoculation, the spores were enumerated by plating serial dilutions on BHIS agar containing 0.1% sodium taurocholate.

Male and female Syrian golden hamsters (*Mesocricetus auratus*, Charles River Laboratories) were housed individually in sterile cages and given a standard rodent diet and water ad libitum. To render the animals susceptible to *C*. *difficile*, one dose of clindamycin (30 mg kg^−1^ of body weight) was administered by oral gavage 7 days prior to inoculation. Hamsters were inoculated with approximately 5,000 spores of a single strain of *C*. *difficile* [84,85]. Uninfected controls treated with clindamycin were included in each experiment. Hamsters were weighed at least daily and monitored for signs of disease (weight loss, lethargy, diarrhea, and wet tail). Fecal samples were collected daily for determination of bacterial burden (see below). Hamsters were considered moribund if they lost 15% or more of their highest weight or if they showed disease symptoms of lethargy, diarrhea, and wet tail. Animals meeting either criterion were euthanized by CO_2_ asphyxiation and thoracotomy. Immediately following euthanasia, animals were necropsied, and cecal contents were obtained for enumeration of total *C*. *difficile* CFU and for subsequent DNA isolation. To enumerate *C*. *difficile* CFU, fecal and cecal samples were weighed, suspended in 1× PBS, heated to 60 °C for 20 minutes to minimize growth of other organisms, and plated on TCCFA [86,87]. *C*. *difficile* CFU were enumerated after 48 hours. Six animals (three male, three female) per *C*. *difficile* strain were tested, and the data were analyzed using GraphPad Prism.

### Semiquantitative PCR and qPCR analysis of DNA from hamster cecums

Hamster cecal contents in PBS were treated with lysozyme and subjected to bead beating to lyse spores. DNA was purified by phenol:chloroform:isopropanol extraction and washed with ethanol. For semiquantitative PCR, 0.5 ng DNA per 50-μL reaction was PCR amplified using 0.5 μM orientation-specific primers to detect the orientation of the *cmr* switch (Cdi6): RT2378-RT2379 for the ON orientation; RT2378-RT2197 for the OFF orientation. For qPCR, 4 ng DNA per 20-μL reaction was used with 0.5 μM orientation-specific primers.

### Sporulation assays

*C*. *difficile* cultures were grown in BHIS medium supplemented with 0.1% taurocholate and 0.2% fructose until the midexponential phase (i.e., OD_600_ of 0.5), and 0.25-mL aliquots were plated onto 70:30 agar supplemented with 2 μg/mL Tm [83]. After 24 hours of growth, ethanol resistance assays were performed as previously described [88,89]. Briefly, the cells were removed from plates after 24 hours (H_24_) and suspended in BHIS medium to an OD_600_ of approximately 1.0. The number of vegetative cells per milliliter was determined by immediately serially diluting and plating the suspended cells onto BHIS. Simultaneously, a 0.5-mL aliquot was added to 0.3 mL of 95% ethanol and 0.2 mL of dH_2_O to achieve a final concentration of 28.5% ethanol, vortexed, and incubated for 15 minutes to eliminate all vegetative cells; ethanol-treated cells were subsequently serially diluted in 1× PBS with 0.1% taurocholate and applied to BHIS plus 0.1% taurocholate plates to determine the total number of spores. After at least 24 hours of growth, CFU were enumerated, and the sporulation frequency was calculated as the total number of spores divided by the total number of viable cells (spores plus vegetative cells). A nonsporulating mutant (MC310; *spo0A*::*erm*) was used as a negative control.

### Germination assays

*C*. *difficile* strains were grown in 500-mL liquid 70:30 medium, and spores were purified for germination studies as previously described, with some modifications [85,90–92]. Cultures in sporulation medium were removed from the anaerobic chamber after 120 hours of anaerobic growth and kept outside of the chamber in atmospheric oxygen overnight. Spore cultures were collected by centrifugation, suspended in water, and frozen for 15 minutes at −80 °C. After thawing, spore suspensions were centrifuged for 10 minutes at 3,200*g* in a swing bucket rotor, and the supernatant was discarded. Spore pellets were washed two times with water and then suspended in 1 mL of a 1× PBS + 1% BSA solution, applied to a 12-mL 50% sucrose bed volume, and centrifuged at 3,200*g* for 20 minutes. The supernatant was decanted, and the spores were checked by phase-contrast microscopy for purity. Sucrose gradients were repeated until the preparations reached a purity of greater than 95%. Spore pellets were then washed three times with 1× PBS + 1% BSA and suspended to an OD_600_ = 3.0. Germination assays were carried out as previously described, with slight modifications [90,91]. After treatment of spores for 30 minutes at 60 °C, spore germination was analyzed in BHIS containing 10 mM taurocholate and 100 mM glycine. The OD_600_ was determined immediately and at various time points during incubation at room temperature. Results are presented as means and standard errors of the means of three independent biological replicates.

### Detection of TcdA by western blot

Rough and smooth isolates as well as *cmrR* and *cmrT* mutants were grown for 24 hours in TY broth and normalized to OD_600_ equal to approximately 1.8 prior to collection. Cells were pelleted, suspended in SDS-PAGE buffer, and boiled for 10 minutes. Samples were then run on 4%–20% Mini-PROTEAN TGX Precast Protein Gels (Bio-Rad) and transferred to a nitrocellulose membrane. Membranes were stained with Ponceau S stain (Sigma Aldrich) to assess sample loading. TcdA was detected as described previously using a mouse α-TcdA primary antibody (Novus Biologicals) and goat anti-mouse IgG conjugated with IR800 (Thermo Fisher) [42].

## Supporting information

S1 Table Strains and plasmids used in this study. (DOCX)

S2 Table Oligonucleotides used in this study. (DOCX)

S1 Data Excel file containing numerical data for all figures and supplemental figures. (XLSX)

S1 Fig Growth of mutant and overexpression strains in vitro. *C*. *difficile* strains were grown in BHIS broth with ATc at 0, 2, or 10 ng/mL for induction. Optical densities (600 nm) over time for R20291 with plasmids for expression of (A) *cmrR* and mutant alleles or (B) *cmrT* and mutant alleles. (C) Optical densities over time for R20291 WT, *cmrR*, and *cmrT* mutants. Shown are means and standard deviations. Data can be found in supplemental file S1 Data. ATc, anhydrotetracycline; BHIS, brain heart infusion plus yeast; *cmr*, colony morphology regulators; WT, wild-type.
(TIF)

S2 Fig The putative response regulators CmrR and CmrT promote the development of rough colonies. *C*. *difficile* R20291 with plasmids for expression of *cmrR*, *cmrT*, or mutant alleles was evaluated for colony morphology on BHIS-1.8% agar with ATc to induce expression (ATc concentrations indicated in ng/mL). Expression of *cmrT* inhibited growth in the presence of 4 ng/μL ATc. ATc, anhydrotetracycline; BHIS, brain heart infusion plus yeast; *cmr*, colony morphology regulators.
(TIF)

S3 Fig Amino acid substitutions of the phosphorylation sites in CmrR and CmrT alter activity. *C*. *difficile* with plasmids for expression of cmrR, cmrT, or mutant alleles, as well as a vector control, were assayed for surface migration on BHIS-1.8% agar 1% glucose (A, B) and for swimming motility through 0.5× BHIS-0.3% agar (C, D). TFP-null (*pilB1*) mutant with vector or *cmrR/cmrT* expression plasmids were included in the surface migration assay (A, B). A nonmotile *sigD* mutant was used as a control for swimming motility experiments (C, D). The media contained ATc at 0, 2, or 10 ng/ml (white, gray, and black bars, respectively) to induce gene expression. (B, D) Expression of *cmrT* inhibited growth at 10 ng/ml and was not included. Shown are the means and standard deviations of the diameters of motile growth after 48 (C, D) or 72 (A, B) hours. **p < 0.005, ^#^*p <* 0.0005, ^+^*p <* 0.0001, two-way ANOVA and Tukey’s posttest. These data are representative of four independent experiments. Data can be found in supplemental file S1 Data. ATc, anhydrotetracycline; BHIS, brain heart infusion plus yeast; *cmr*, colony morphology regulators; *sigD*, sigma factor D gene; TFP, type IV pilus.
(TIF)

S4 Fig CmrR and CmrT do not regulate flagellum or TFP gene expression. *C*. *difficile* with vector or *cmrR*/*cmrT* expression plasmids were grown for 48 hours in 0.5× BHIS-0.3% agar to express flagellar genes. Bacteria were recovered and cultured in TY broth with inducer (10 ng/mL ATc for vector and pCmrR; 2 ng/mL ATc for pCmrT). Samples were collected at the midexponential phase for RNA extraction and qRT-PCR analysis. The data were analyzed using the ΔΔCt method with *rpoC* as the reference gene and no ATc as the control condition. Shown are the means and standard deviations of three biological replicates. Data can be found in supplemental file S1 Data. ATc, anhydrotetracycline; BHIS, brain heart infusion plus yeast; *cmr*, colony morphology regulators; qRT-PCR, quantitative real-time PCR; TFP, type IV pilus; TY, Tryptone Yeast.
(TIF)

S5 Fig The *cmrT* mutant is defective in cell elongation and chaining. (A) R20291 WT, Δ*cmrR*, and Δ*cmrT* cultures were spotted and grown on BHIS 1.8% agar 1% glucose for 72 hours. Cells from the colony edge were collected, Gram stained, and imaged at ×60 magnification. Shown are representative images. (B) Quantification of cell lengths in Gram stain images from (A). At least two images from two biological replicates were used. The lengths of more than 514 cells per strain were measured using ImageJ and normalized to the average WT cell length. Means and standard deviations are shown. *p < 0.0001, one-way ANOVA. Data can be found in supplemental file S1 Data. (C) Representative images of the colony edges of WT, Δ*cmrR*, Δ*cmrT*, and *pilB1*::*erm*. Cultures were spotted and grown on BHIS 1.8% agar 1% glucose for 72 hours and imaged at ×2 (top) and ×20 (bottom) magnification. BHIS, brain heart infusion plus yeast; *cmr*, colony morphology regulators; *erm*, erythromycin resistance cassette; WT, wild-type.
(TIF)

S6 Fig The *cmrR* mutant exhibits an increase in biofilm formation. R20291 smooth and rough isolates and the *cmrR*, and *cmrT* mutants were grown in BHIS 1% glucose 50 mM sodium phosphate buffer for 24 hours in 24-well polystyrene plates. Adhered biofilms were washed and quantified using a crystal violet staining assay. The means of four to five technical replicates were normalized to values for the R20291 rough isolate and combined from two independent experiments. *p < 0.05, one-way ANOVA with Dunnett’s posttest. Data can be found in supplemental file S1 Data. BHIS, brain heart infusion plus yeast; *cmr*, colony morphology regulators.
(TIF)

S7 Fig *cmrR* and *cmrT* mutants are not defective in sporulation, germination, or toxin production. (A) Sporulation of R20291 rough and smooth isolates and the *cmrR* and *cmrT* mutants after 24 hours on 70:30 agar. Sporulation is expressed as a percentage of viable spores versus total cells and then normalized to values obtained for the rough isolate. (B) Germination of spores over time after addition of the germinants taurocholic acid and glycine. (A, B) No statistically significant differences were observed using a one-way ANOVA, *N =* 3 biological replicates. (C) TcdA levels in bacterial lysates were assessed after 24 hours of growth in TY medium by western blot. Ponceau S staining was used to determine equal sample loading. (D) Quantification of TcdA western blots for four biological replicates. Intensity of the TcdA bands for each was normalized to intensity of Ponceau S staining per lane. Values were then normalized to the intensity for the smooth isolate. Shown is a representative image. *p < 0.05, one-way ANOVA with Tukey’s posttest. (E) qRT-PCR analysis *tcdA* mRNA levels in strains overexpressing *cmrR*, *cmrT*, or vector control. Bacteria were cultured in BHIS broth with inducer (10 ng/mL ATc for vector and pCmrR; 2 ng/mL ATc for pCmrT). The data were analyzed using the ΔΔCt method with *rpoC* as the reference gene and R20291 with vector as the control condition. Shown are the means and standard deviations of four to six biological replicates. No statistically significant differences were observed using a one-way ANOVA. Data can be found in supplemental file S1 Data. ATc, anhydrotetracycline; BHIS, brain heart infusion plus yeast; *cmr*, colony morphology regulators; qRT-PCR, quantitative real-time PCR; TAG, taurocholic acid and glycine germinants; TY, Tryptone Yeast.
(TIF)

## Abbreviations

ATc: anhydrotetracycline
ATCC: American Type Culture Collection
BHIS: brain heart infusion plus yeast
c-di-GMP: cyclic diguanosine monophosphate
CFU: colony-forming unit
CmrRST: colony morphology regulators RST
CwpV: cell wall protein V
DccA: diguanylate cyclase A
*erm*: erythromycin resistance cassette
GTPase: guanosine triphosphatase
NCBI: National Center for Biotechnology Information
qPCR: quantitative PCR
qRT-PCR: quantitative real-time PCR
Rho: Ras homologous
RM: restriction-modification
SEM: scanning electron microscopy
SigD: sigma factor D
TCCFA: taurocholate cycloserine cefoxtin fructose agar
TFP: type IV pilus
WT: wild-type
