# Supplementary material for: Phase variation of a signal transduction system controls Clostridioides difficile colony morphology, motility, and virulence
Source: PLoS Biol. 2019 Oct 28;17(10):e3000379. doi: 10.1371/journal.pbio.3000379 (PMC6837544; doi:10.1371/journal.pbio.3000379)
Supplement: S1 Table — (DOCX) [file pbio.3000379.s001.docx]

**Phase variation of a signal transduction system controls *Clostridioides difficile* colony morphology, motility, and virulence**

Elizabeth M. Garrett, Ognjen Sekulovic, et al.

**SUPPLEMENTAL MATERIAL**

**S1 Table. Strains and plasmids used in this study**

| **Lab Notation** | **Strain/Plasmid Name** | **Description** | **Reference** |
| --- | --- | --- | --- |
|  | *Escherichia coli* DH5α | F- φ80*lacZ*ΔM15 Δ(*lacZY*A-*argF*)U169 *recA1* *endA1* *hsdR*17(rκ -, mκ+) *phoA supE44 thi*-1 *gyrA96* *relA1* λ- *tonA* | Invitrogen  (1) |
| RT270 | *Escherichia coli* HB101(pRK24) | *E. coli* used in conjugations with *C. difficile*, Ap^R^, Cm^R^ | (2) |
| RT1124 | *C. difficile* 630 | Ribotype 012 strain (Genbank Accession # AM180355) |  |
| RT273 | *C. difficile* R20291 | Ribotype 027 strain (Genbank Accession # FN545816) | (3) |
| RT1065 | *C. difficile* UK1 | Ribotype 027 strain | (4, 5) |
| RT1125 | *C. difficile* VPI 10463 | Ribotype 003 strain | (6) |
| RT1357 | *C. difficile* ATCC BAA-1875 | Ribotype 078 strain | ATCC |
| RT1358 | *C. difficile* ATCC 43598 | Ribotype 017 strain | ATCC, (7) |
| RT526 | R20291 pMC-Pcpr | R20291 with pMC-Pcpr, nisin inducible | (8) |
| RT527 | R20291 pDccA | R20291 with pMC-Pcpr::*dccA*, nisin inducible | (8) |
| RT528 | R20291 pPdcA-EAL | R20291 with pMC-Pcpr::EAL, nisin inducible | (9) |
| RT539 | R20291 pDccA^mut^ | R20291 with pMC-Pcpr::*dccA*^mut^, nisin inducible | (8) |
| RT2196 | R20291 pRPF185 | R20291 with pRPF185, ATc inducible | This work |
| RT2085 | R20291 pCmrR | R20291 pRPF185::*cmrR*, ATc inducible | This work |
| RT2086 | R20291 pCmrR-D52E | R20291 pRPF185::*cmrR-*D52E, ATc inducible | This work |
| RT2087 | R20291 pCmrR-D52A | R20291 pRPF185::*cmrR*-D52A, ATc inducible | This work |
| RT2107 | R20291 pCmrT | R20291 pRPF185::*cmrT*, ATc inducible | This work |
| RT2201 | R20291 pCmrT-D53A | R20291 pRPF185::*cmrT*-D53A, ATc inducible | This work |
| RT1566 | R20291 *sigD* | R20291 *sigD::ermB*, Targetron insertion | (10) |
| RT2111 | R20291 *sigD* pMC-Pcpr | R20291 *sigD::ermB* with pMC-Pcpr | This work |
| RT2112 | R20291 *sigD* pMC-pDccA | R20291 *sigD::ermB* with pMC-Pcpr::*dccA* | This work |
| RT2113 | R20291 *sigD* pPdcA-EAL | R20291 *sigD::ermB* with pMC-Pcpr::EAL | This work |
| RT947 | R20291 *pilB* | R20291 *pilB::ermB,* Targetron insertion | (8) |
| RT2177 | R20291 *pilB* pMC-Pcpr | R20291 *pilB::ermB* with pMC-Pcpr | This work |
| RT2178 | R20291 *pilB* pDccA | R20291 *pilB::ermB* with pMC-Pcpr::*dccA* | This work |
| RT2179 | R20291 *pilB* pMC-Pcpr | R20291 *pilB::ermB* with pMC-Pcpr::EAL | This work |
| RT2180 | R20291 *pilB* pCmrR | R20291 *pilB::ermB* with pRPF185::*cmrR* | This work |
| RT2197 | R20291 *pilB* pRPF185 | R20291 *pilB::ermB* with pRPF185 | This work |
| RT2204 | R20291 *pilB* pCmrT | R20291 *pilB::ermB* with pRPF185::*cmrT* | This work |
| RT2256 | R20291 Δ*cmrR* | R20291 with in-frame deletion of *cmrR* | This work |
| RT2257 | R20291 Δ*cmrT* | R20291 with in-frame deletion of *cmrT* | This work |
| RT2267 | R20291 Δ*cmrR* pRPF185 | R20291 Δ*cmrR* with pRPF185 | This work |
| RT2268 | R20291 Δ*cmrR* pCmrR | R20291 Δ*cmrR* with pRPF185::*cmrR* | This work |
| RT2269 | R20291 Δ*cmrT* pRPF185 | R20291 Δ*cmrT* with pRPF185 | This work |
| RT2270 | R20291 Δ*cmrT* pCmrT | R20291 Δ*cmrT* with pRPF185::*cmrT* | This work |
| RT2296 | R20291 Δ*cmrR* Δ*cmrT* | R20291 with in-frame deletions of *cmrR* and *cmrT* | This work |
| MC310 | R20291 *spo0A* | R20291 *spo0A::ermB* | (11) |
| RT399 | pMC-P*_cpr_* | pMC123 with nisin-inducible *cpr* promoter | (9) |
| RT402 | pDccA | pMC-Pcpr::*dccA* (CD630_14200), encodes DGC | (9) |
| RT529 | pDccA^mut^ | pMC-Pcpr::*dccA*^mut^ (AADEF), encodes inactive DGC | (9) |
| RT404 | pPdcA-EAL | pMC-Pcpr::*pdcA*-EAL | (12) |
| RT709 | pRPF185 | Contains ATc-inducible P*tet* promoter | (13) |
| RT2073 | pCmrR | pRPF185::*cmrR* | This work |
| RT2074 | pCmrR-D52E | pRPF185::*cmrR*-D52E | This work |
| RT2075 | pCmrR-D52A | pRPF185::*cmrR*-D52A | This work |
| RT2106 | pCmrT | pRPF185::*cmrT* | This work |
| RT2200 | pCmrT-D53A | pRPF185::*cmrT*-D53A | This work |

**References**

1. Hanahan D. 1983. Studies on transformation of *Escherichia coli* with plasmids. J Mol Biol 166:557-80.

2. McBride SM, Sonenshein AL. 2011. Identification of a genetic locus responsible for antimicrobial peptide resistance in *Clostridium difficile*. Infect Immun 79:167-176.

3. Stabler RA, He M, Dawson L, Martin M, Valiente E, Corton C, Lawley TD, Sebaihia M, Quail MA, Rose G, Gerding DN, Gibert M, Popoff MR, Parkhill J, Dougan G, Wren BW. 2009. Comparative genome and phenotypic analysis of *Clostridium difficile* 027 strains provides insight into the evolution of a hypervirulent bacterium. Genome Biol 10:R102.

4. Killgore G, Thompson A, Johnson S, Brazier J, Kuijper E, Pepin J, Frost EH, Savelkoul P, Nicholson B, van den Berg RJ, Kato H, Sambol SP, Zukowski W, Woods C, Limbago B, Gerding DN, McDonald LC. 2008. Comparison of seven techniques for typing international epidemic strains of *Clostridium difficile*: restriction endonuclease analysis, pulsed-field gel electrophoresis, PCR-ribotyping, multilocus sequence typing, multilocus variable-number tandem-repeat analysis, amplified fragment length polymorphism, and surface layer protein A gene sequence typing. J Clin Microbiol 46:431-7.

5. Sorg JA, Sonenshein AL. 2010. Inhibiting the initiation of *Clostridium difficile* spore germination using analogs of chenodeoxycholic acid, a bile acid. J Bacteriol 192:4983-90.

6. Sullivan NM, Pellett S, Wilkins TD. 1982. Purification and characterization of toxins A and B of *Clostridium difficile*. Infect Immun 35:1032-40.

7. Depitre C, Delmee M, Avesani V, L'Haridon R, Roels A, Popoff M, Corthier G. 1993. Serogroup F strains of *Clostridium difficile* produce toxin B but not toxin A. J Med Microbiol 38:434-41.

8. Purcell EB, McKee RW, Bordeleau E, Burrus V, Tamayo R. 2015. Regulation of Type IV Pili Contributes to Surface Behaviors of Historical and Epidemic Strains of *Clostridium difficile*. J Bacteriol 198:565-577.

9. Purcell EB, McKee RW, McBride SM, Waters CM, Tamayo R. 2012. Cyclic diguanylate inversely regulates motility and aggregation in *Clostridium difficile*. J. Bacteriol 194:3307-3316.

10. Anjuwon-Foster BR, Tamayo R. 2017. A genetic switch controls the production of flagella and toxins in *Clostridium difficile*. PLoS Genet 13:e1006701.

11. Edwards AN, Nawrocki KL, McBride SM. 2014. Conserved oligopeptide permeases modulate sporulation initiation in *Clostridium difficile*. Infect Immun 82:4276-4291.

12. Purcell EB, McKee RW, Courson DS, Garrett EM, McBride SM, Cheney RE, Tamayo R. 2017. A nutrient-regulated cyclic diguanylate phosphodiesterase controls *Clostridium difficile* biofilm and toxin production during stationary phase. Infect Immun 85:pii: e00347-17.

13. Fagan RP, Fairweather NF. 2011. *Clostridium difficile* has two parallel and essential Sec secretion systems. The J Biol Chem 286:27483-27493.
