# Supplementary material for: Phase variation of a signal transduction system controls Clostridioides difficile colony morphology, motility, and virulence
Source: PLoS Biol. 2019 Oct 28;17(10):e3000379. doi: 10.1371/journal.pbio.3000379 (PMC6837544; doi:10.1371/journal.pbio.3000379)
Supplement: S2 Table — (DOCX) [file pbio.3000379.s002.docx]

**The phase-variable two-component system CmrRST regulates colony morphology, host colonization, and virulence in *Clostridium difficile***

Elizabeth M. Garrett, Ognjen Sekulovic, et al.

**SUPPLEMENTAL MATERIAL**

**S2 Table. Oligonucleotides used in this study**

| **Lab Notation** | **Primer name** | **Sequence** **(5’ to 3’)**^a^ |
| --- | --- | --- |
| R2175 | qPCR_FlgSwit-ON | GTTTTCTTACCAAAGTGATACATTATTATATTAATG |
| R2176 | qPCR_FlgSwit-OFF | CATTAATATAATAATGTATCACTTTGGTAAGAAAAC |
| R2177 | qPCR_FlgSwit-REV | GCTATTGTCTGACTTCTTAAATTAGTTGCAT |
| R2213 | LCF801 | GGTAAGTTTGATTTTTATGTTAATGAATTG |
| R2214 | LCF714 | CAGTTTGTGCACTAGCTATGCCTGC |
| R2215 | LCF796 | CGCAATTATTTGTTTTTCATATGGATAAAATTGG |
| R2216 | LCF797 | GATTTTTATGTTAATGAATTGTTATAAAAAACATGG |
| R2261 | CDR20291_0685-IEq1 | GTTAAAAATTTAAGATATCTTTTCAGTATAATGGA |
| R2262 | CDR20291_0685-IEq2 | CATTTCTAAGAAATATCCTAACATAAAAACAAAA |
| R2263 | CDR20291_0685-IEq3 | CGATTACACTACAGAATTAGAATGTCAATG |
| R2264 | CDR20291_0963-IEq1 | GTAAATTAAGATGTATTTCATTTCTCAAAAATATCCT |
| R2265 | CDR20291_0963-IEq2 | GTAAAGTTTATAAAATCTGAAAAGCTCAAGA |
| R2266 | CDR20291_0963-IEq3 | GCTTTTATCGCAAGTTTGTTTTAAATGAC |
| R2270 | CDR20291_3128-IEq1 | GGAGATATATGGAGTTAGTGGTGCAA |
| R2271 | CDR20291_3128-IEq2 | CTAGCCAATAGACAAGTTTCTAGAAAAATA |
| R2272 | CDR20291_3128-IEq3 | GAACAATTCTTGAATATTGTATTGAACATTAAGA |
| R2273 | RpoAqF | TCATTACCAGGTGTAGCAGTGAATGC |
| R2274 | RpoAqR | GATAGAGCATGGTCCTTGAGCTTCT |
| R2378 | OS 196 | GTACAGAAGTTACCCAGAAGCTTGT |
| R2379 | OS 197 | TCCCCGCAATGGATGTTTTTTAATTCATC |
| R2380 | OS 198 | TCCCAATTTAAATGTAGAGGTCATCAAT |
| R2527 | 3128_F | TACGAGCTCCTTGAGATTATGATTAAAATACCTTTG |
| R2528 | 3128_R | TACGGATCCCAGTATCTCACTTATGGTACAAACTTATAT |
| R2529 | 3128pm_glu_F | GTATAATTTTGGAAATTTCATTGCC |
| R2530 | 3128pm_glu_R | GGCAATGAAATTTCCAAAATTATAC |
| R2531 | 3128pm_ala_F | GTATAATTTTGGCTATTTCATTGCC |
| R2532 | 3128pm_ala_R | GGCAATGAAATAGCCAAAATTATAC |
| R2533 | 3126_F | TACGAGCTCTAATATAAAAGAATAATGATATTTGGGAGTG |
| R2534 | 3126_R | TACGGATCCCGTTAGCATTTCACATTTATAAC |
| R2535 | 3126pm_ala_F | TTTAGCAATAATTCTAACTGATGGTG |
| R2536 | 3126pm_ala_R | CACCATCAGTTAGAATTATTGCTAAA |
|  | OS158 | gtgttttttgttaccctaagtttAGTAATAGTATCAAGAGAAGAAG |
|  | OS163 | agattatcaaaaaggagtttCCACATCTGCCAAGAATTTTTTAC |
|  | OS266 | agtatctcacTACACCACTCCATTCAAAG |
|  | OS267 | gagtggtgtaGTGAGATACTGTAATTAATAAATAGTTCTTG |
|  | OS268 | ttttttgttaccctaagtttGCCATCCATGTATCCTATC |
|  | OS269 | ttaatatatttaTATAATCACTCCCAAATATCATTATTC |
|  | OS271 | agattatcaaaaaggagtttTGTCAAATCTTGTAACCAAC |
|  | OS283 | agtgattataGAAAATTATAACAATAAGAGGAGC |
| R850 | rpoCqF | CTAGCTGCTCCTATGTCTCACATC |
| R851 | rpoCqR | CCAGTCTCTCCTGGATCAACTA |
| R856 | CD0245qF | GCAACTAATCTAAGAAGTCAGACAATAGC |
| R857 | CD0245qR | AGGCATAGCATCATTTAGTGTTTCTTC |
| R858 | CD0229qF | CAAGTGTATCAAATATGAGCGATGAA |
| R859 | CD0229qR | TTATCCTCGCATCTCCTCTATCATT |
| R1063 | fliCqF | TACAAGTTGGAGCAAGTTATGGAAC |
| R1064 | fliCqR | GTTGTTATACCAGCTGAAGCCATTA |
| R930 | qRTpilA1F | TGGCAGTTCCAGCTTTATTTAGTAAT |
| R931 | qRTpilA1R | AAGATAATGCTGCACTCTTAACTGA |
| R852 | CD0663qF | GGAGAAGTCAGTGATATTGCTCTTG |
| R853 | CD0663qR | CAGTGGTAGAAGATTCAACTATAGCC |
| R2298 | CDR_3128qF1 | AAGAAAAAGTTCGGGGATTTTTAGC |
| R2299 | CDR_3128qR1 | CGCTGAAAACTTTAACACATTAGGA |
| R2537 | 3126_qF | GACAAGGATAATTGCC |
| R2538 | 3126_qR | CCATCACCATCAGTTAG |

^a^ Restriction sites used for cloning are underlined
