# Supplementary figures and images for: Phase variation of a signal transduction system controls Clostridioides difficile colony morphology, motility, and virulence
Source: PLoS Biol. 2019 Oct 28;17(10):e3000379. doi: 10.1371/journal.pbio.3000379 (PMC6837544; doi:10.1371/journal.pbio.3000379)

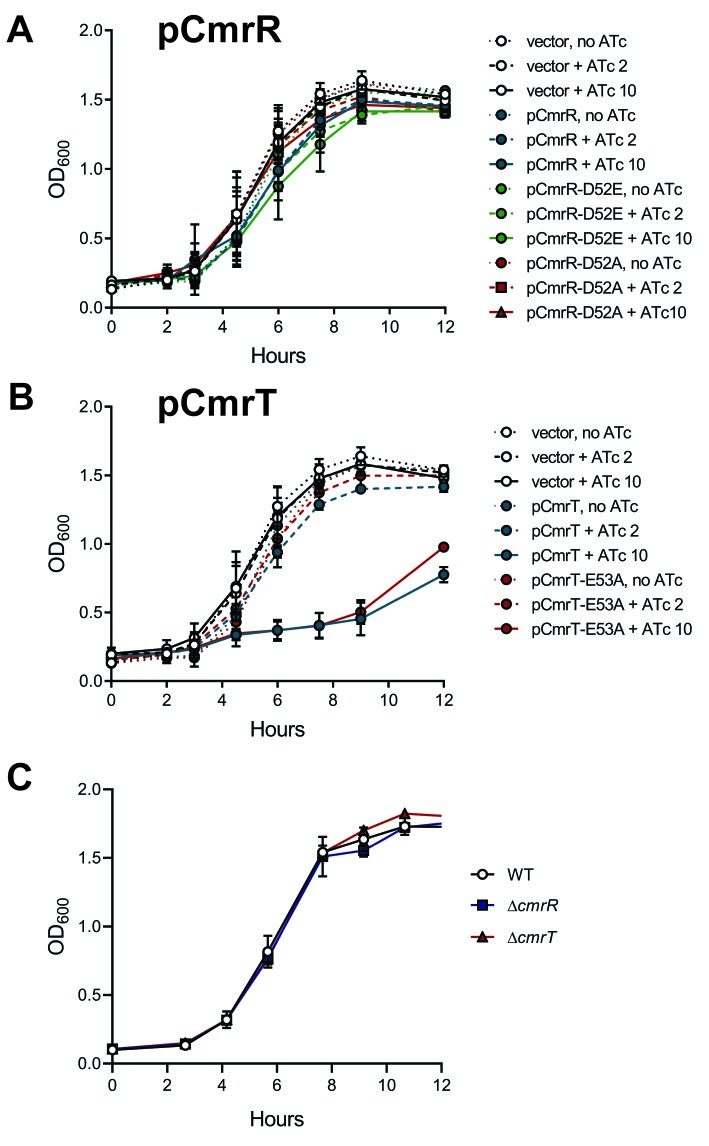

Supplement: S1 Fig — C. difficile strains were grown in BHIS broth with ATc at 0, 2, or 10 ng/mL for induction. Optical densities (600 nm) over time for R20291 with plasmids for expression of (A) cmrR and mutant alleles or (B) cmrT and mutant alleles. (C) Optical densities over time for R20291 WT, cmrR, and cmrT mutants. Shown are means and standard deviations. Data can be found in supplemental file S1 Data. ATc, anhydrotetracycline; BHIS, brain heart infusion plus yeast; cmr, colony morphology regulators; WT, wild-type. (TIF) [file pbio.3000379.s004.tif]

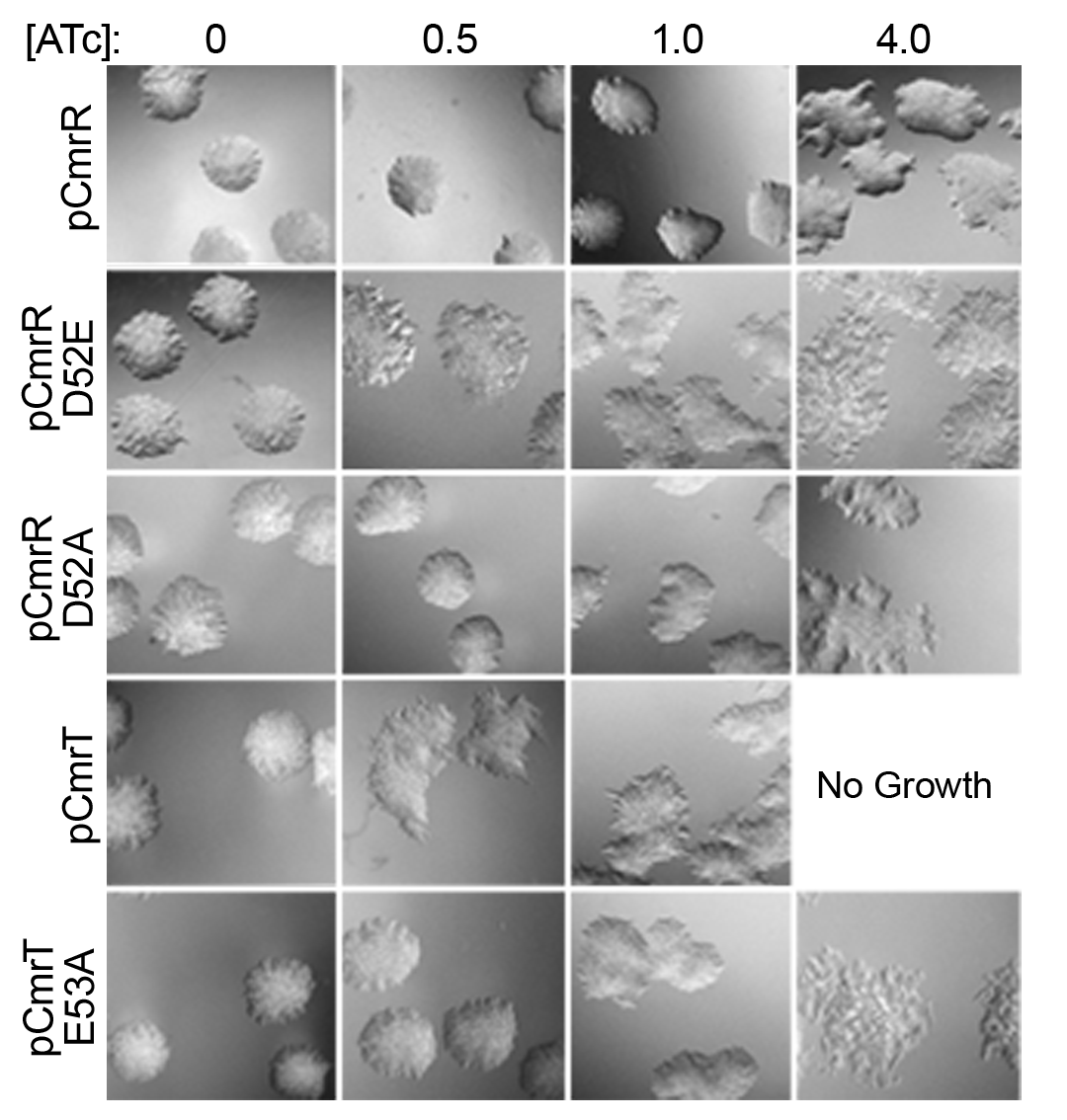

Supplement: S2 Fig — C. difficile R20291 with plasmids for expression of cmrR, cmrT, or mutant alleles was evaluated for colony morphology on BHIS-1.8% agar with ATc to induce expression (ATc concentrations indicated in ng/mL). Expression of cmrT inhibited growth in the presence of 4 ng/μL ATc. ATc, anhydrotetracycline; BHIS, brain heart infusion plus yeast; cmr, colony morphology regulators. (TIF) [file pbio.3000379.s005.tif]

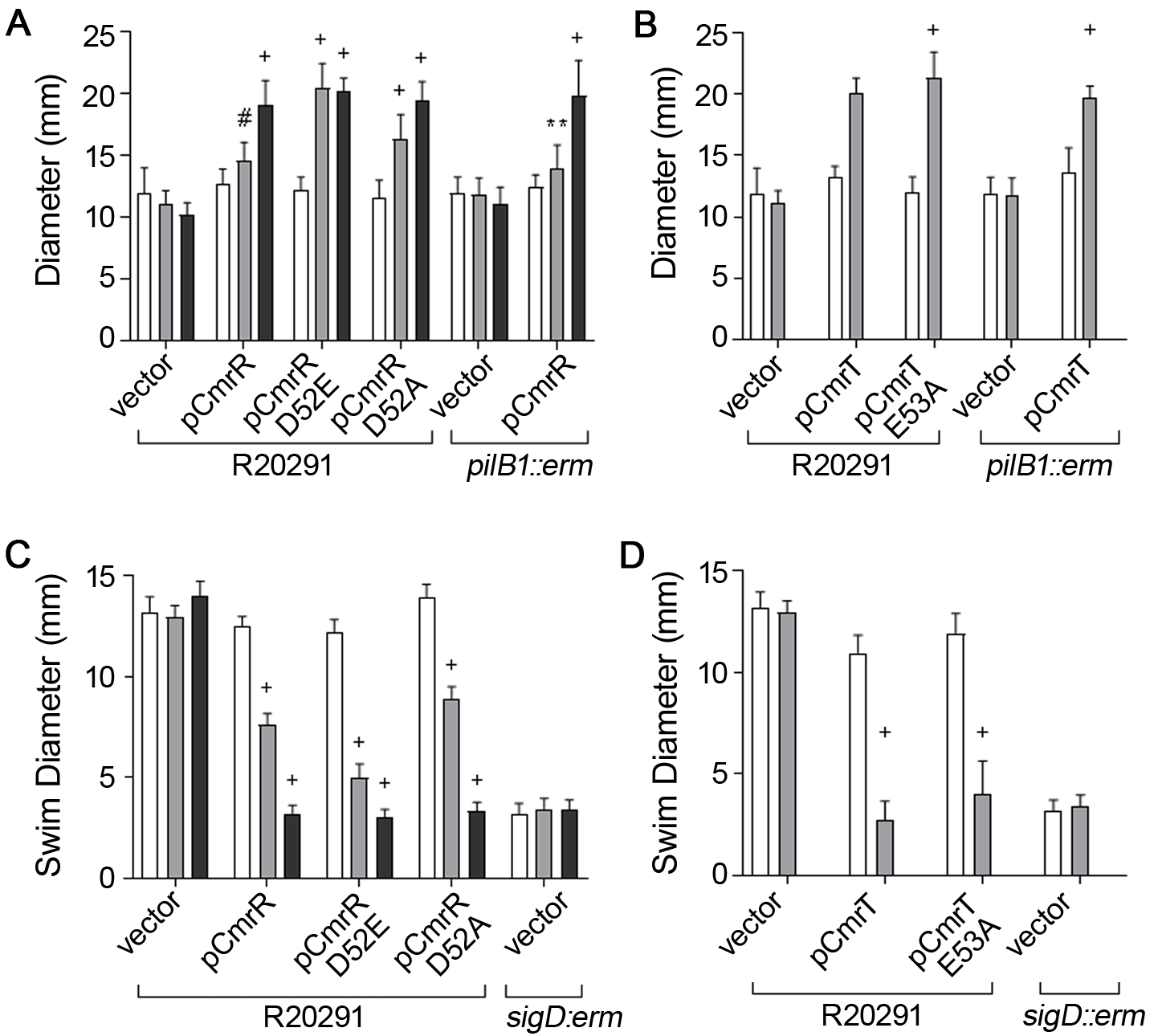

Supplement: S3 Fig — C. difficile with plasmids for expression of cmrR, cmrT, or mutant alleles, as well as a vector control, were assayed for surface migration on BHIS-1.8% agar 1% glucose (A, B) and for swimming motility through 0.5× BHIS-0.3% agar (C, D). TFP-null (pilB1) mutant with vector or cmrR/cmrT expression plasmids were included in the surface migration assay (A, B). A nonmotile sigD mutant was used as a control for swimming motility experiments (C, D). The media contained ATc at 0, 2, or 10 ng/ml (white, gray, and black bars, respectively) to induce gene expression. (B, D) Expression of cmrT inhibited growth at 10 ng/ml and was not included. Shown are the means and standard deviations of the diameters of motile growth after 48 (C, D) or 72 (A, B) hours. **p < 0.005, #p < 0.0005, +p < 0.0001, two-way ANOVA and Tukey’s posttest. These data are representative of four independent experiments. Data can be found in supplemental file S1 Data. ATc, anhydrotetracycline; BHIS, brain heart infusion plus yeast; cmr, colony morphology regulators; sigD, sigma factor D gene; TFP, type IV pilus. (TIF) [file pbio.3000379.s006.tif]

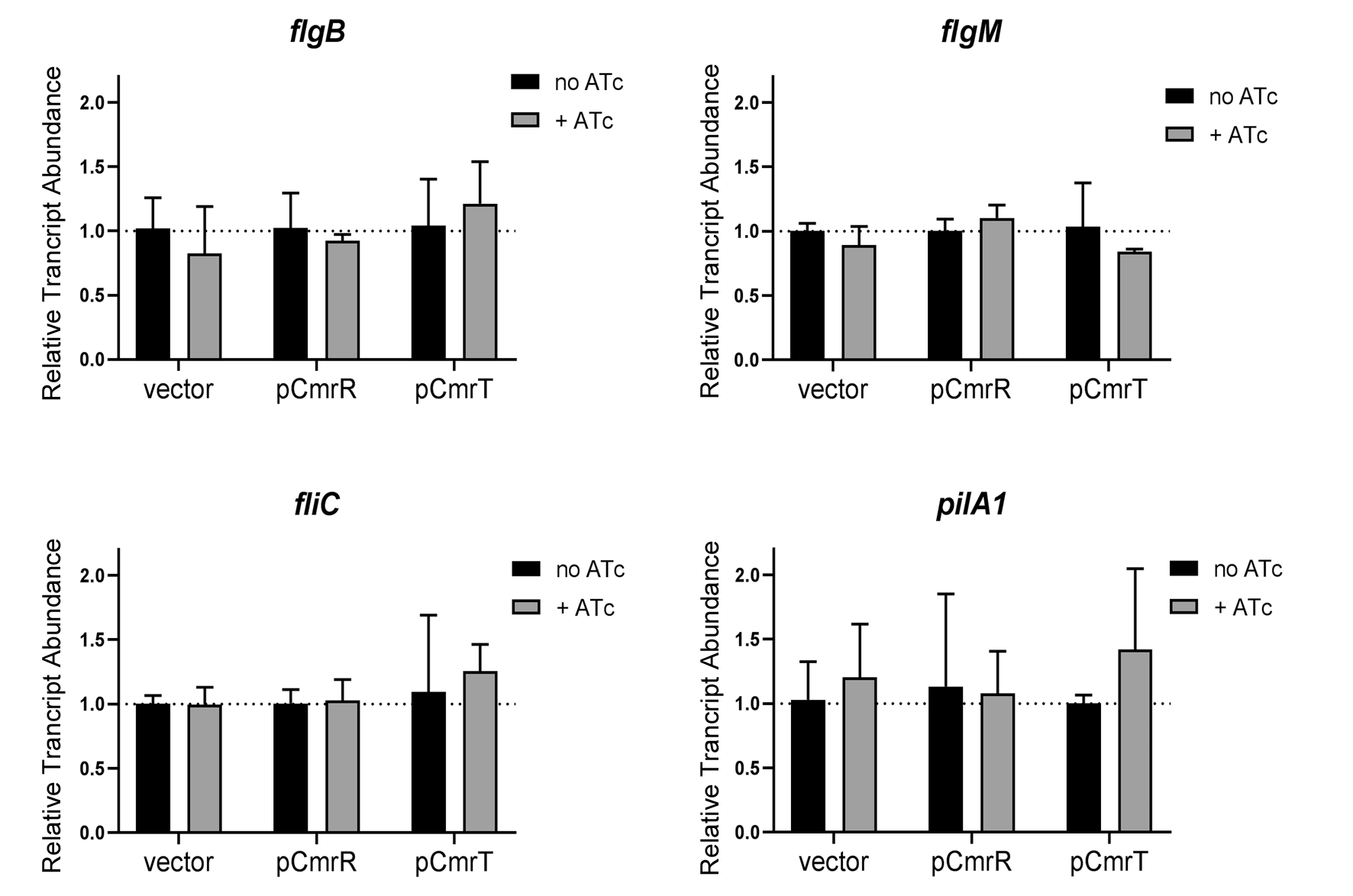

Supplement: S4 Fig — C. difficile with vector or cmrR/cmrT expression plasmids were grown for 48 hours in 0.5× BHIS-0.3% agar to express flagellar genes. Bacteria were recovered and cultured in TY broth with inducer (10 ng/mL ATc for vector and pCmrR; 2 ng/mL ATc for pCmrT). Samples were collected at the midexponential phase for RNA extraction and qRT-PCR analysis. The data were analyzed using the ΔΔCt method with rpoC as the reference gene and no ATc as the control condition. Shown are the means and standard deviations of three biological replicates. Data can be found in supplemental file S1 Data. ATc, anhydrotetracycline; BHIS, brain heart infusion plus yeast; cmr, colony morphology regulators; qRT-PCR, quantitative real-time PCR; TFP, type IV pilus; TY, Tryptone Yeast. (TIF) [file pbio.3000379.s007.tif]

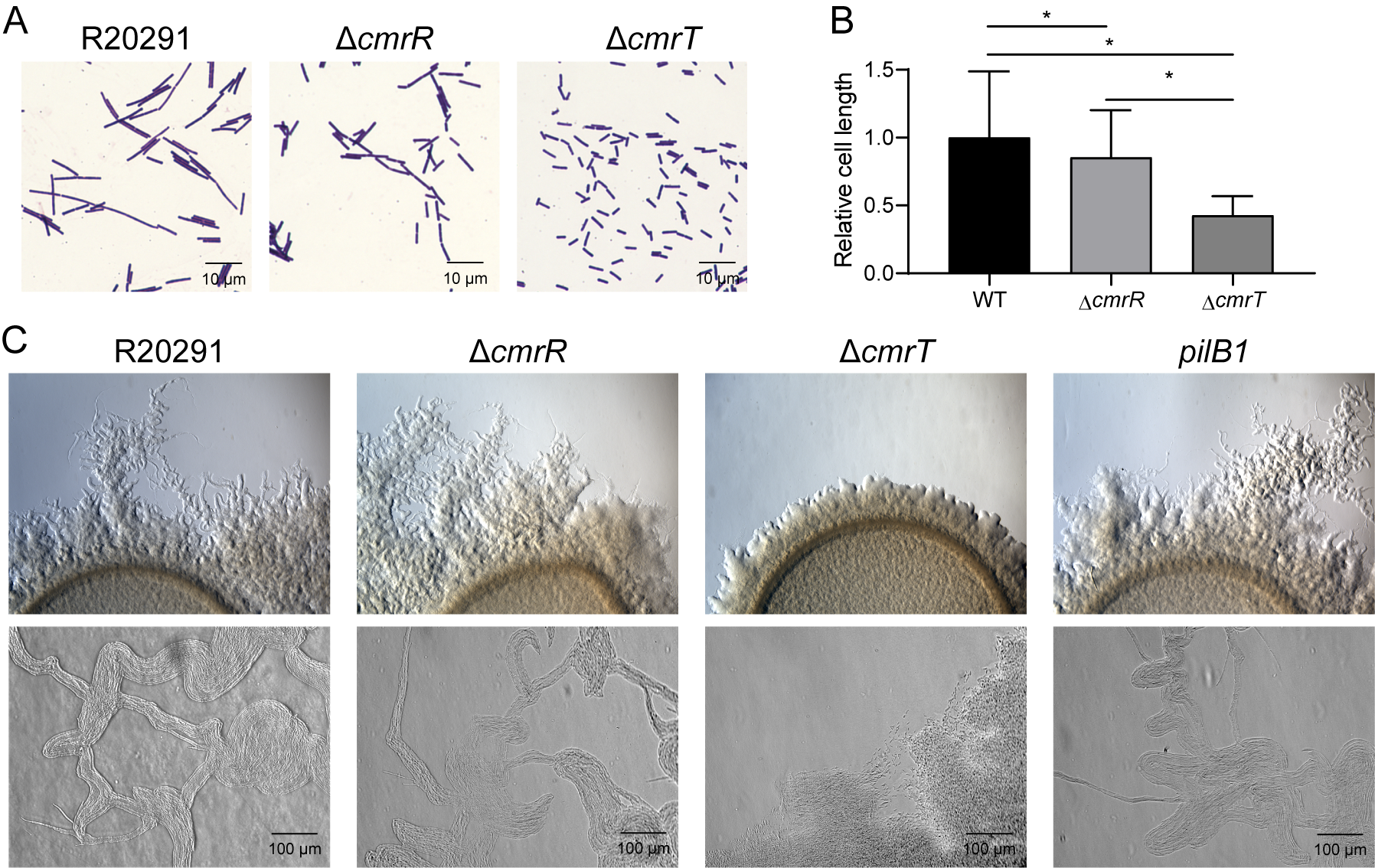

Supplement: S5 Fig — (A) R20291 WT, ΔcmrR, and ΔcmrT cultures were spotted and grown on BHIS 1.8% agar 1% glucose for 72 hours. Cells from the colony edge were collected, Gram stained, and imaged at ×60 magnification. Shown are representative images. (B) Quantification of cell lengths in Gram stain images from (A). At least two images from two biological replicates were used. The lengths of more than 514 cells per strain were measured using ImageJ and normalized to the average WT cell length. Means and standard deviations are shown. *p < 0.0001, one-way ANOVA. Data can be found in supplemental file S1 Data. (C) Representative images of the colony edges of WT, ΔcmrR, ΔcmrT, and pilB1::erm. Cultures were spotted and grown on BHIS 1.8% agar 1% glucose for 72 hours and imaged at ×2 (top) and ×20 (bottom) magnification. BHIS, brain heart infusion plus yeast; cmr, colony morphology regulators; erm, erythromycin resistance cassette; WT, wild-type. (TIF) [file pbio.3000379.s008.tif]

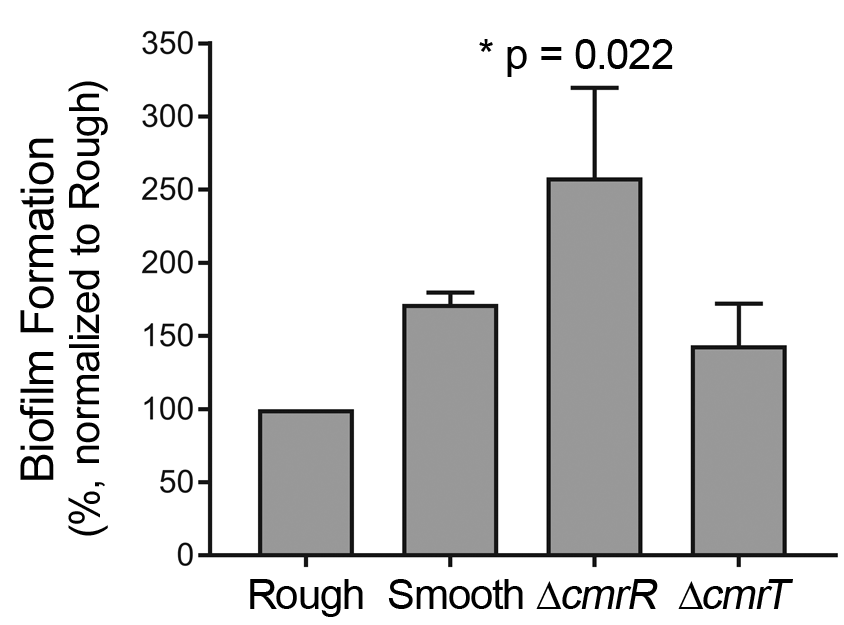

Supplement: S6 Fig — R20291 smooth and rough isolates and the cmrR, and cmrT mutants were grown in BHIS 1% glucose 50 mM sodium phosphate buffer for 24 hours in 24-well polystyrene plates. Adhered biofilms were washed and quantified using a crystal violet staining assay. The means of four to five technical replicates were normalized to values for the R20291 rough isolate and combined from two independent experiments. *p < 0.05, one-way ANOVA with Dunnett’s posttest. Data can be found in supplemental file S1 Data. BHIS, brain heart infusion plus yeast; cmr, colony morphology regulators. (TIF) [file pbio.3000379.s009.tif]

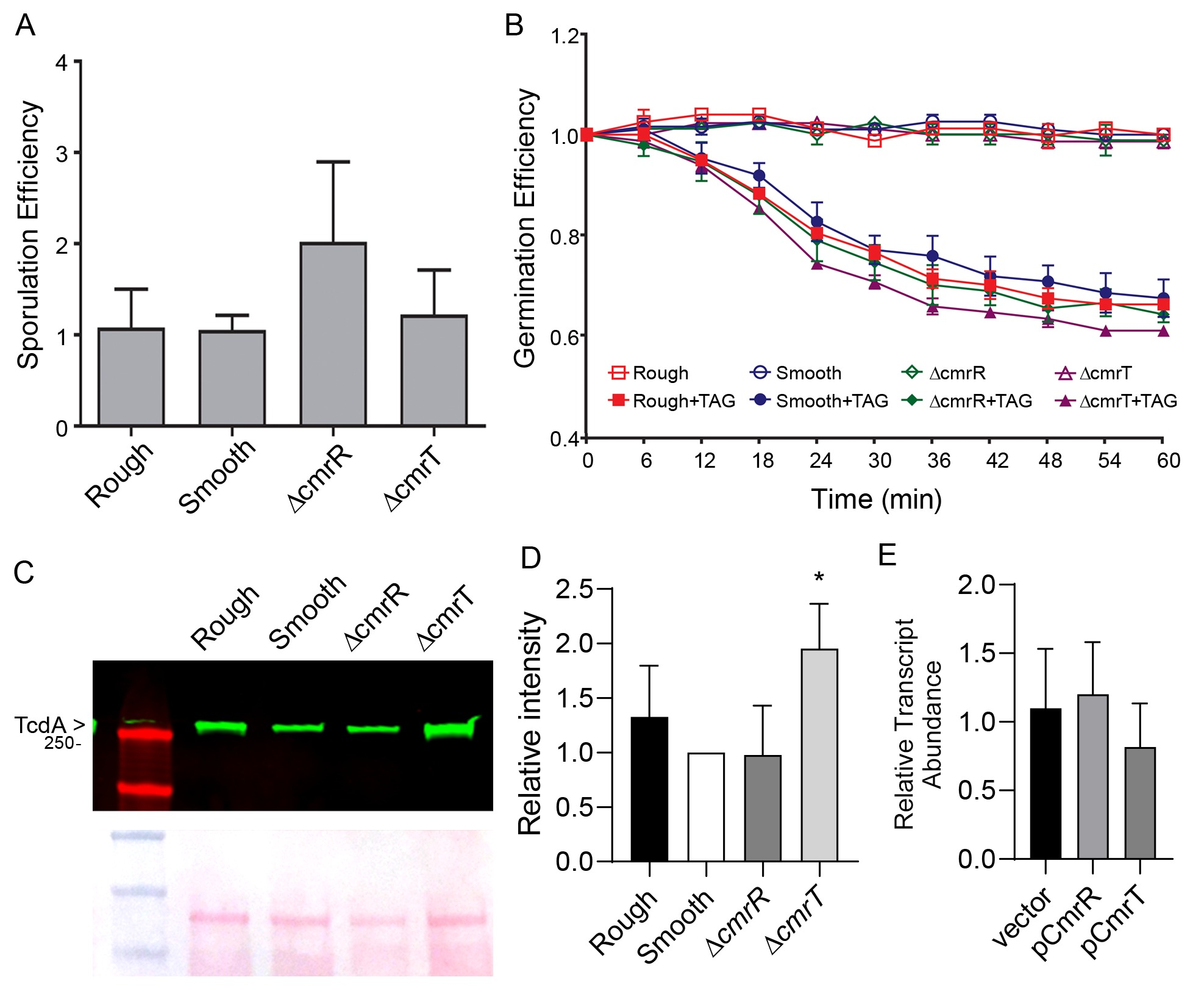

Supplement: S7 Fig — (A) Sporulation of R20291 rough and smooth isolates and the cmrR and cmrT mutants after 24 hours on 70:30 agar. Sporulation is expressed as a percentage of viable spores versus total cells and then normalized to values obtained for the rough isolate. (B) Germination of spores over time after addition of the germinants taurocholic acid and glycine. (A, B) No statistically significant differences were observed using a one-way ANOVA, N = 3 biological replicates. (C) TcdA levels in bacterial lysates were assessed after 24 hours of growth in TY medium by western blot. Ponceau S staining was used to determine equal sample loading. (D) Quantification of TcdA western blots for four biological replicates. Intensity of the TcdA bands for each was normalized to intensity of Ponceau S staining per lane. Values were then normalized to the intensity for the smooth isolate. Shown is a representative image. *p < 0.05, one-way ANOVA with Tukey’s posttest. (E) qRT-PCR analysis tcdA mRNA levels in strains overexpressing cmrR, cmrT, or vector control. Bacteria were cultured in BHIS broth with inducer (10 ng/mL ATc for vector and pCmrR; 2 ng/mL ATc for pCmrT). The data were analyzed using the ΔΔCt method with rpoC as the reference gene and R20291 with vector as the control condition. Shown are the means and standard deviations of four to six biological replicates. No statistically significant differences were observed using a one-way ANOVA. Data can be found in supplemental file S1 Data. ATc, anhydrotetracycline; BHIS, brain heart infusion plus yeast; cmr, colony morphology regulators; qRT-PCR, quantitative real-time PCR; TAG, taurocholic acid and glycine germinants; TY, Tryptone Yeast. (TIF) [file pbio.3000379.s010.tif]
